# Targeting AKT1-E17K and the PI3K/AKT Pathway with an Allosteric AKT Inhibitor, ARQ 092

**DOI:** 10.1371/journal.pone.0140479

**Published:** 2015-10-15

**Authors:** Yi Yu, Ronald E. Savage, Sudharshan Eathiraj, Justin Meade, Michael J. Wick, Terence Hall, Giovanni Abbadessa, Brian Schwartz

**Affiliations:** 1 Biology, ArQule, Inc., Burlington, Massachusetts, United States of America; 2 Preclinical Development and Clinical Pharmacology, ArQule, Inc., Burlington, Massachusetts, United States of America; 3 Preclinical Research, South Texas Accelerated Research Therapeutics, San Antonio, Texas, United States of America; 4 Clinical Development, ArQule, Inc., Burlington, Massachusetts, United States of America; H.Lee Moffitt Cancer Center & Research Institute, UNITED STATES

## Abstract

As a critical component in the PI3K/AKT/mTOR pathway, AKT has become an attractive target for therapeutic intervention. ARQ 092 and a next generation AKT inhibitor, ARQ 751 are selective, allosteric, pan-AKT and AKT1-E17K mutant inhibitors that potently inhibit phosphorylation of AKT. Biochemical and cellular analysis showed that ARQ 092 and ARQ 751 inhibited AKT activation not only by dephosphorylating the membrane-associated active form, but also by preventing the inactive form from localizing into plasma membrane. In endometrial PDX models harboring mutant AKT1-E17K and other tumor models with an activated AKT pathway, both compounds exhibited strong anti-tumor activity. Combination studies conducted in *in vivo* breast tumor models demonstrated that ARQ 092 enhanced tumor inhibition of a common chemotherapeutic agent (paclitaxel). In a large panel of diverse cancer cell lines, ARQ 092 and ARQ 751 inhibited proliferation across multiple tumor types but were most potent in leukemia, breast, endometrial, and colorectal cancer cell lines. Moreover, inhibition by ARQ 092 and ARQ 751 was more prevalent in cancer cell lines containing PIK3CA/PIK3R1 mutations compared to those with wt-PIK3CA/PIK3R1 or PTEN mutations. For both ARQ 092 and ARQ 751, PIK3CA/PIK3R1 and AKT1-E17K mutations can potentially be used as predictive biomarkers for patient selection in clinical studies.

## Introduction

AKT (Protein kinase B, PKB) is a serine/threonine kinase that belongs to the AGC family of kinases. It was discovered as the mouse leukemia virus AKT 8 oncogene and in humans is encoded by three genes AKT1, 2 and 3 [1]. The AKT family members affect a wide range of biological functions including cell proliferation and growth, metabolism, protein synthesis, migration, angiogenesis, and anti-apoptotic ability [2, 3]. In order for AKT to modulate these functions, activation is essential. Full activation of AKT requires translocation to the plasma membrane followed by phosphorylation of both the threonine 308 and serine 473 residues which are regulated by multiple components of the well-known PI3K/AKT/mTOR pathway. In this pathway, PI3K phosphorylates phosphatidylinositol (3,4) bisphosphate (PIP2) to phosphatidylinositol (3,4,5) triphosphate (PIP3). The pleckstrin homology (PH) domains of both PI3K-dependent kinase 1 (PDK1) and AKT bind to PIP3 (located at the plasma membrane) thus allowing PDK1 to phosphorylate the activation loop of AKT at Thr 308. The activation process is completed with the phosphorylation of AKT at the C-terminal Ser 473 of the hydrophobic motif which occurs via the mTORC2/rictor complex [4, 5]. Once activated, AKT phosphorylates downstream target proteins including PRAS40, GSK3, FOXO, and BAD [2]. Additionally, AKT also suppresses the inhibitory effect via phosphorylation of TSC1 and TSC2, thereby activating mTORC1 and ultimately leading to phosphorylation of 4E-BP1 and S6K [6, 7].

Dysregulation of PI3K/AKT/mTOR signaling has been linked to various diseases including cancer and overgrowth syndromes [8–13]. The tumor suppressor PTEN (phosphatase and tensin homolog deleted on chromosome ten) negatively regulates PI3K activity by dephosphorylating PIP3 to PIP2, which in turn deactivates AKT [14]. Hence, in cancers where PTEN is inactivated via a mutation or deletion, AKT is activated [15, 16]. These activations occur frequently in prostate and endometrial cancers as well as glioblastoma and melanoma [15]. AKT activation can also occur via constitutive activation of PI3K through receptor tyrosine kinase activation and mutations predominantly in the *PIK3CA* gene. Both *PIK3CA* activating and *PTEN* loss mutations have been found to occur frequently in breast, endometrial, and colon cancers with reported incidence rates ranging from 6–35%, 22–54%, and from 9 to 54%, respectively [13, 15]. Consequently, AKT represents an attractive target for therapeutic intervention and as such there are many AKT inhibitors in pre-clinical and clinical development [11, 17–22].

Activating mutations in the PH domain of AKT1, AKT2 and AKT3 which constitutively activate the kinase (E17K) have been reported in a variety of different oncologic and non-oncologic diseases [23–26]. The AKT1-E17K mutant protein has been shown to have increased cell membrane localization and was capable of transforming Rat1 fibroblasts and ultimately inducing leukemia in mice [24]. This mutation has been reported in numerous cancers including breast, colorectal, ovarian and endometrial cancers with the highest incidence being in breast (6–8%), colorectal cancer (2–6%) and meningioma (6%) [23, 24, 27–36]. Though occurring with low frequency rates in cancer, this mutation has been found to be much more prevalent in patients with Proteus syndrome (PS), a rare condition characterized by the overgrowth of bone, fat, skin, and other connective tissues. Lindhurst et al. recently reported that of 29 patients with PS, 26 (90%) carried the AKT1-E17K mutation [10]. Other non-oncologic diseases in which AKT2-E17K and AKT3-E17K mutations cause hypoglycemia and hemimegalencephaly have also been reported [25, 26].

We report data on ARQ 092 and ARQ 751 (S1 Fig) as highly potent and selective allosteric inhibitors of AKT and demonstrate that they are active both in biochemical assays and in cells which contain wild-type (WT) or mutant AKT. In addition, both compounds were shown to potently suppress tumor growth and the AKT pathway in xenograft models harboring either WT or AKT1-E17K, with ARQ 751 consistently demonstrating superior potency compared to ARQ 092. These data support the clinical advancement of both compounds not only in cancers where AKT mutants including AKT1-E17K are prevalent, but also in rare overgrowth diseases like PS where the AKT1-E17K mutant potentially drives disease progression.

## Methods

### Reagents

ARQ 092 was synthesized at ArQule, Inc. (Burlington, MA). ARQ 751 was synthesized at Daiichi Sankyo RD Novare Co. Ltd (Daiichi Sankyo Ltd, Japan). MK-2206 was synthesized by SAI Life Sciences (Hyderabad, India) or at Daiichi Sankyo RD Novare Co. Ltd. GDC-0068 was purchased from Selleckchem (Houston, TX). Paclitaxel was purchased from Bristol-Myers Squibb (New York, NY) and trastuzumab was purchased from Chugai Pharmaceutical Co. (Tokyo, Japan). Trametinib was purchased from LC Laboratories (Woburn, MA). AN3CA, BT-474, ZR-75-1, MDA-MB-453, NCI-H1650, NIH 3T3 and 293T cells were purchased from American Tissue and Culture Collection (Manassas, VA). KU-19-19 cells were purchased from Creative Bioarray (Shirley, NY). KPL-4 cells were kindly provided by Dr. J. Kurebayashi (Kawasaki Medical School, Okayama, Japan) [37]. Human PDGF-BB was purchased from PeproTech (Rocky Hill, NJ). Methyl cellulose 400cP solution was purchased from Wako Pure Chemical Industries Ltd (Osaka, Japan) and DMA was purchased from Sigma Aldrich (St Louis, MO). All experimental mice were purchased from Taconic USA (Hudson, NY) or CLEA Japan, Inc. (Tokyo, Japan). All other reagents were of the highest grade and obtained from standard suppliers.

### Biochemical IC_50_ determination and kinase profiling

Biochemical IC_50_ determination for ARQ 092, ARQ 751, MK-2206, and GDC-0068 against full-length AKT1, AKT2, and AKT3 was performed at Reaction Biology (Malvern, PA). Compounds were tested in a 10-dose mode with 3-fold serial dilutions starting at 1 μM in the presence of 10 μM ATP and the IC_50_ was determined. Curve fits were performed where the enzyme activities at the highest concentration of compounds were less than 65% of the enzyme activity (relative to DMSO controls).

Kinase profiling was performed for ARQ 092 against 303 kinases and for ARQ 751 against 245 kinases at 5 μM at Carna Biosciences, Inc. (Kobe, Japan). The % inhibition was determined relative to DMSO control. For ARQ 092, a confirmatory IC_50_ value was determined for any kinase that had greater than 50% inhibition at 5 μM.

### Cell Culture Conditions

All cancer cell lines were grown in EMEM, DMEM or RPMI-1640 (Life Technologies Corporation, Carlsbad, CA) supplemented with 10% FBS. 293T and NIH 3T3 cells were cultured in DMEM with or without GlutaMAX, respectively, supplemented with 10% FBS. All cell cultures were maintained at 37°C in a humidified atmosphere containing 5% CO_2_.

### Expression DNA Constructs and Transfection

Construction of the expression vector was performed at BPS Biosciences (San Diego, CA). WT and E17K mutant forms of the human *AKT1* cDNA were fused with green fluorescent protein (GFP) in the c-terminus and cloned into the pcDNA expression vector, generating pcDNAAKT1-WT-GFP and pcDNAAKT1-E17K-GFP. AKT1-WT and AKT1-E17K were validated by sequencing.

Cells were transiently transfected with pcDNAAKT1-WT-GFP or pcDNAAKT1-E17K-GFP using Lipofectamine 2000 (Life Technologies, Inc., Grand Island, NY).

### Western Blot Analyses

Cells (MDA-MB-453: 1.5x10^6^; NCI-H1650: 1x10^6^; KU-19-19: 0.7x10^6^) were plated into 6 well plates, left overnight, and then treated with full media containing different concentrations (0, 0.012, 0.037, 0.11, 0.33, and 1 μM) of AKT inhibitors (ARQ 092, ARQ 751, MK-2206, GDC-0068) for 2 hours. Cells were treated under designated conditions and lysates were extracted. Proteins were resolved from extracts using SDS-PAGE followed by immunoblotting. P-AKT(T308), p-AKT(S473), total AKT, p-PRAS40(T246), total PRAS40, pGSK3b(S9), pAS160(S318), pFOXO1(T24)/3a(T32), pBAD(S136), pS6(S235/S236), p4E-BP1(S65), pERK(T202/Y204), and total ERK, were assessed using the corresponding primary antibodies (Cell Signaling Technology, Beverly, MA). Anti-actin was purchased from Sigma Aldrich (St. Louis, MO). HRP conjugated secondary antibodies were purchased from Cell Signaling Technology. Immunoblotting detection was performed using ECL reagent (GE Healthcare, Piscataway, NJ).

Tumor tissues were ground in mortars with liquid nitrogen and lysed. Proteins extracted from tumor tissues were resolved using E-PAGE (Life Technologies Corporation) followed by immunoblotting. P-AKT(T308), p-AKT(S473), total AKT, p-PRAS40(T246) and β-Actin were assessed with corresponding primary antibodies (Cell Signaling Technology). Infrared dye conjugated secondary antibodies (LI-COR Biosciences, Lincoln, NE) were used as secondary antibodies. Immunoblotting detection was performed with a LI-COR scanner (LI-COR Biosciences). The intensity of the bands was quantitated using the accompanying software. The data were expressed as percent inhibition in comparison to vehicle.

### Intrinsic tryptophan fluorescence quench assay

The binding of ARQ 092, ARQ 751, MK-2206, and GDC-0068 to AKT1-WT and AKT1-E17K was followed by monitoring intrinsic tryptophan fluorescence quench. Inhibitors were serially diluted (two-fold) with buffer solution (25 mM Tris, pH 7.5, 0.1 M NaCl and 2% DMSO) in 384 well plates. AKT1 or AKT1-E17K (100 nM) was added to each well and diluted to a final volume of 50 μl with buffer. The resulting mixtures were allowed to equilibrate for 30 minutes at room temperature. The change in intrinsic emission was measured on a Tecan microplate reader (Tecan, Mannedorf, Switzerland) at an excitation wavelength of 280 nm and emission wavelength of 345 nm. The fluorescence at 345 nm was plotted versus the inhibitor concentration, and the data were fit to a quadratic equation using nonlinear regression. The following expression relating observed fluorescence (*F*) to inhibitor concentration [*I*_0_] was used to obtain the binding constant for AKT inhibitors: (K_D_^app^), *F* = *F*_0_ + (Δ*F*/*P*_0_) [(*K*_D_^app^ + [*P*_0_] + [*I*_0_])—the square root of [(*K*_D_^app^ + [*P*_0_] + [*I*_0_])^2^–4[*P*_0_][*I*_0_]]]/2, where *P*_0_ is the initial protein concentration (constant), *K*_D_ is the dissociation constant, Δ*F* is the total change in fluorescence, and *I*_0_ is the inhibitor concentration. Each titration experiment represents an average obtained from four independent experiments.

### AKT1 Plasma Membrane Translocation

Cells (15,000 cells per chamber) were plated into a Nunc Lab-Tek Chambered Coverglass System (Thermo Fisher Scientific, Waltham, MA) containing full media without antibiotics. Next, the cells were transfected with AKT-GFP plasmids using 1 μL Lipofectamine 2000 (0.81 μg of AKT1-E17K-GFP and 0.6 μg of AKT1-WT-GFP) in the presence of 30 μL Opti-MEM. After 48 hours, cells were incubated in serum free media for an additional 24 hours and then treated with or without inhibitor (1 μM ARQ 092, ARQ 751, MK-2206, or GDC-0068) for 2 hours. Next, the cells were stimulated with PDGF (PeproTech, Rocky Hill, NJ) at 50 ng/ml for 10 minutes. Cells were washed with PBS and then fixed with 200 μl paraformaldehyde fixation buffer (3% paraformaldehyde, 2% sucrose in PBS, pre-warmed at 60–70°C). After washing with PBS, cells were blocked and permeabolized with 0.1% TX-100 + 1% BSA in PBS for 15 minutes at room temperature. Next, 200 μl DAPI (1 μg/ml in PBS) was used to counterstain the nuclei at room temperature in darkness for 15 minutes. Samples were then rinsed with cold PBS and stored at 4°C until imaging. Images were captured using an Olympus inverted epi-fluorescent microscope (Olympus Corporation of America, Center Valley, PA) with a 60x oil immersion objective.

### OncoPanel Analysis

Anti-proliferative activity of ARQ 092 and ARQ 751 was assessed using OncoPanel 240 (Eurofins, St. Charles, MO). Cells were seeded into 384-well plates and incubated in a humidified atmosphere of 5% CO_2_ at 37°C. Compounds were added 24 hours post cell seeding. At the same time, a time zero untreated cell plate was generated. Compounds were serially diluted 3.16-fold and assayed over a range of 10 concentrations in buffer containing 0.1% DMSO. Automated fluorescence microscopy was carried out using an IN Cell Analyzer 1000 (GE Healthcare, Marlborough, MA), and images were collected with a 4X objective. After a 72 hour incubation period, cells were fixed and stained with fluorescently labeled antibodies and nuclear dye to determine relative cell number. Twelve bit tiff images were acquired using the IN Cell Analyzer 1000 3.2 and analyzed with Developer Toolbox 1.6 software (GE Healthcare). GI_50_ values were calculated using nonlinear regression to fit data to a sigmoidal 4 point, 4 parameter One-Site dose response model, where: y (fit) = A + [(B–A)/(1 + ((C/x) ^ D))]. Curve-fitting, GI_50_ calculations and report generation are performed using a custom data reduction engine MathIQ based software (AIM).

### Mutation Analysis

Mutation analysis was performed based on information from the COSMIC database (http://cancer.sanger.ac.uk/cancergenome/projects/cosmic/). Cells with GI_50_<1 μM were designated as sensitive while cells with GI_50_>1 μM were designated as resistant. Mutation analysis was performed based on ArQule’s internal database derived from the Catalogue of Somatic Mutations in Cancer (COSMIC) v41. The Fisher’s exact test was applied. A p<0.05 was considered statistically significant.

### In Vivo Studies

All experimental procedures were performed according to the Guide for the Care and Use of Laboratory Animals and approved by ArQule’s Institutional Animal Care and Use Committee, according to the in-house guidelines of the Institutional Animal Care and Use Committee of Daiichi Sankyo and approved by the general manager of the Nonclinical Research Center according to the institutional guideline for animal studies, or according to Guide for the Care and Use of Laboratory Animals and approved by the START Institutional Animal Care and Use Committee.

For acute pharmacodynamic studies, AN3CA cells (5x10^6^) or BT-474 cells (10x10^6^) were inoculated subcutaneously into female NCr *nu/nu* mice three weeks prior to ARQ 092 administration. ARQ 092 was orally dosed at either 100 mg/kg or 200 mg/kg for AN3CA bearing mice (n = 8) and 200 mg/kg for BT-474 bearing mice (n = 4 for Vehicle; n = 5 for ARQ 092). Tumor tissues were collected after 1 or 8 hours for AN3CA or 2 hours for BT-474.

For efficacy studies, AN3CA cells (5x10^6^) were inoculated into female NCr *nu/nu* mice. When tumors reached a size of 250 mm^3^, mice were randomly grouped into 5 groups. Vehicle (10% DMA in water), 50 mg/kg, 100 mg/kg, or 150mg/kg of ARQ 092 was dosed orally daily while 200 mg/kg was dosed every other day. In ARQ 751 xenograft studies, ARQ 751 was orally dosed daily at 5, 10, 20, 40, 80, or 120 mg/kg. KPL-4 cells (1.5x10^7^) were inoculated subcutaneously into female nude mice (BALB/cAJc1-*nu/nu*). When the average size of the tumor reached over 100 mm^3^, mice were randomly divided into 6 groups: vehicle (0.5% w/v% methyl cellulose 400cP solution) and ARQ 092 at 10, 20, 40, 80, or 120 mg/kg. For each group, ARQ 092 was orally dosed daily for 8 days. ZR-75-1 tumor fragments were inoculated into female NOD/scid mice. When tumors reached a size of 300 mm^3^, untreated control, or ARQ 092 at 20 mg/ kg or 40 mg/kg was orally dosed on a schedule of 5 days on, 2 days off and 4 days on.

The endometrial PDX tumor model (ST1061) was developed from a 43 year old African American female with chemotherapy naïve endometrial adenocarcinoma harboring an AKT1-E17K mutation. In this study, athymic nude mice (Charles River Labs) were subcutaneously implanted with tumor fragments and the study initiated seven days later at a mean tumor volume of approximately 200 mm^3^. Mice (n = 10 per dose group) were treated with vehicle or drug at doses of 50, 75, or 100 mg/kg for ARQ 092 and 25, 50, or 75 mg/kg for ARQ 751. At these levels, animals were dosed once daily for 20 days on a schedule of 5 days of dosing followed by 2 days with no dosing.

A melanoma PDX tumor model (ST052C) that contained BRAF V600E and PIK3CA (H1047R) mutations and exhibited acquired vemurafenib-resistance (originating from a 60 year old Caucasian male) was used in this study. Athymic nude mice (Charles River Laboratories, Wilmington, MA) were subcutaneously implanted with tumor fragments and the study was initiated seven days later when the mean tumor volume was approximately 140 mm^3^. Mice (n = 5 per dose group) were treated with vehicle, ARQ 092 at 100 mg/kg, trametinib at 3 mg/kg, and ARQ 092 and trametinib combined. Initially, mice were dosed with ARQ 092 in a schedule of 5 days on and 2 days off for ARQ 092 and trametinib daily. After 13 days, the dosing schedule for trametinib was reduced to a schedule of 5 days on and 2 days off.

For combination studies with trastuzumab, female nude mice (BALB/cAJc1-*nu/nu*) bearing KPL-4 tumors were orally dosed with ARQ 092 at 60 mg/kg daily for 12 days with trastuzumab being intravenously administrated at a dose of 15 mg/kg every three days. Paclitaxel was dosed, intravenously at a dose of 15 mg/kg every 6 days.

The tumor length and width (mm) were measured using a digital caliper. The estimated tumor volume (mm^3^) was calculated according to the following formula: 0.5 x (tumor length) x (tumor width)^2^, and reported as the mean ± SEM. The tumor growth inhibition (TGI) was determined and the vehicle group was designated as 100% tumor growth.

### Immunohistochemical Analyses

All tissue specimens were fixed for 16–24 hours in 10% neutral buffered formalin (NBF) and embedded in paraffin. Immunohistochemistry (IHC) was performed on tumor tissue sections (5 μm thick) with anti-human antibodies p-AKT(S473) and p-PRAS40(T246). After deparaffinization and rehydration of the tissue sections, antigen retrieval was conducted in a decloaking chamber for 30 minutes at 95°C, followed by 10 seconds at 90°C using citrate buffer (pH 6, Poly Scientific, Bay Shore, NY). IHC was performed using a Labvision 480 autostainer (Thermo Fisher Scientific). Rabbit on Rodent HRP-Polymer (Biocare Medical, Concord, CA) was used as a secondary antibody. Primary antibodies were diluted in 1% BSA in Tris-buffered Saline-Tween 20 (Sigma Aldrich). Tissue sections were incubated with primary antibody for 30 minutes and then with polymer for 25 minutes at ambient temperature. The reagent 3,3’-diaminobenzidine (DAB) was used as the chromogen and the slides were counterstained with hematoxylin after IHC. Images were captured using an Aperio Scanscope (Aperio, Vista, CA).

### Statistical Analysis

In order to determine the correlation between mutations and ARQ 092 or ARQ 751 sensitivity, the Fisher exact test was applied. A p<0.05 was considered statistically significant.

For *in vivo* studies, all statistical analyses were performed using an unpaired *t* test. Data were presented as mean ± SEM. A p<0.05 was considered statistically significant.

## Results

### ARQ 092 and ARQ 751 bind the inactive form of AKT1 and AKT1-E17K mutant and inhibit phosphorylation

**Fig 1:**
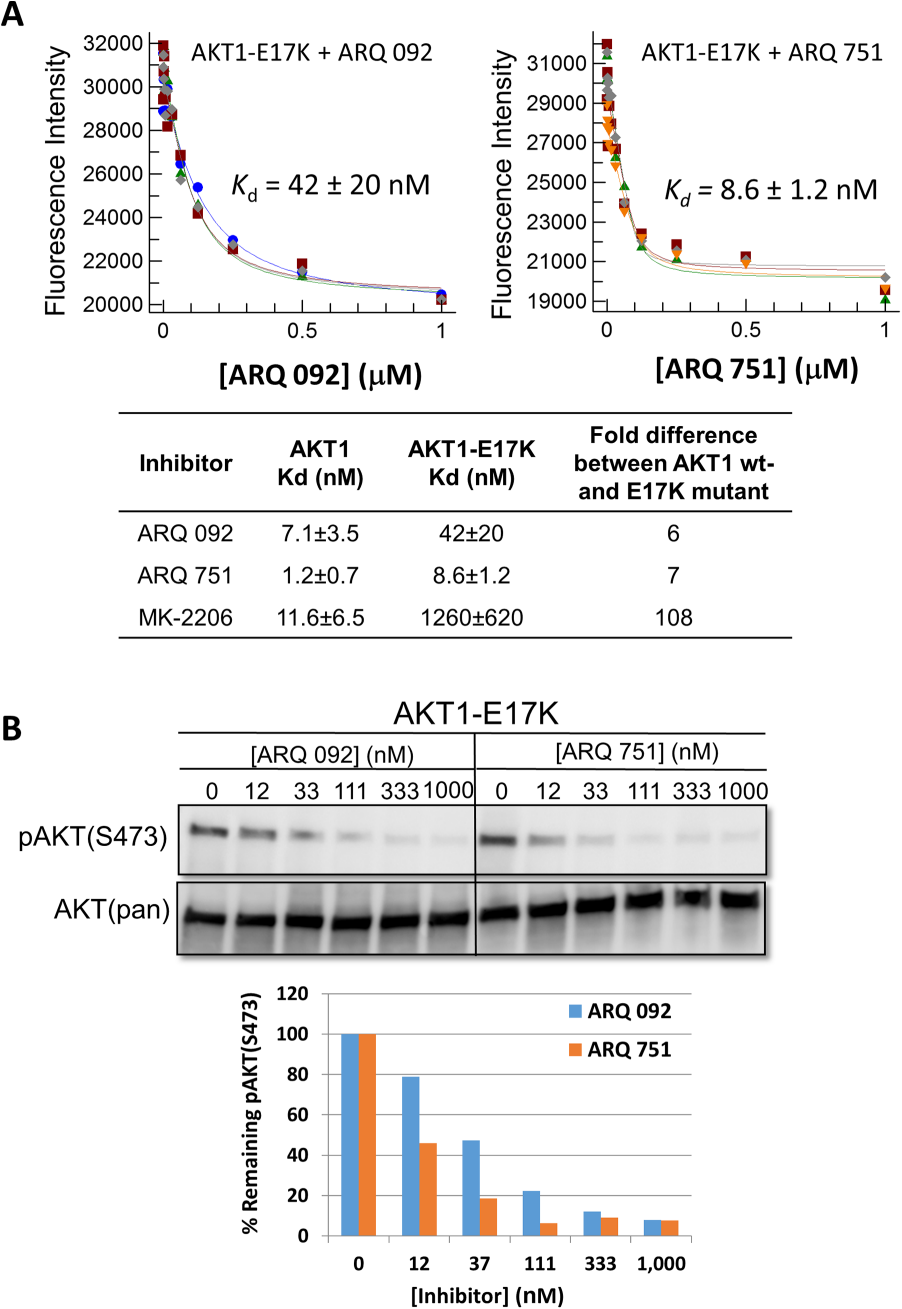
Determination of *K*_*d*_ values for the allosteric inhibitors ARQ 092, ARQ 751, and MK-2206 and pathway inhibition by ARQ 092 and ARQ 751 in transfected 293T cells. (A) The *K*_*d*_ values of ARQ 092, ARQ 751, and MK-2206 were determined against AKT-WT and AKT1-E17K. (B) 293T cells were transiently transfected with pcDNA-E17K-GFP and then treated with various concentrations of ARQ 092 or ARQ 751 for 2 hours. pAKT(S473) and total AKT were assessed. Densitometry analysis was performed and relative pAKT(S473) level was shown as percentage (%) of pAKT/total AKT.

The binding of ARQ 092 and ARQ 751 to the unphosphorylated, full-length form of AKT and AKT1-E17K mutant was determined by measuring the change in intrinsic tryptophan fluorescence. The data showed that both ARQ 092 and ARQ 751 bind potently to the WT and E17K mutant forms of AKT1. For AKT1-WT, *K*_*d*_ values were 7.1 and 1.2 nM for ARQ 092 and ARQ 751, respectively (Fig 1A). For the AKT1-E17K mutant, *K*_*d*_ values were 42 and 8.6 nM for ARQ 092 and ARQ 751, respectively. These data clearly demonstrate that the allosteric inhibitors ARQ 092 and ARQ 751 potently bind to AKT1-E17K. As expected, the reference compound MK-2206 showed only weak binding to AKT1-E17K in this assay, however, previous reports have shown that MK-2206 is still capable of pathway inhibition in cells harboring this mutation [38].

In order to determine whether ARQ 092 and ARQ 751 were able to inhibit phosphorylation of AKT1-E17K, we constructed green fluorescent protein (GFP) tagged WT and E17K mutant forms of AKT1 (pcDNAAKT-WT-GFP and pCDNAAKT1-E17K-GFP) and transiently introduced them into 293T cells. We then assessed phosphorylation levels of pAKT(S473) after cells were treated with different concentrations of ARQ 092 or ARQ 751. Both ARQ 092 and ARQ 751 significantly suppressed phosphorylation of GFP tagged endogenous WT (S2 Fig) and E17K mutant forms of AKT1 (Fig 1B). These data demonstrate that ARQ 092 and ARQ 751 bind to both WT and E17K mutant forms of AKT and decrease levels of their phosphorylation.

**Fig 2:**
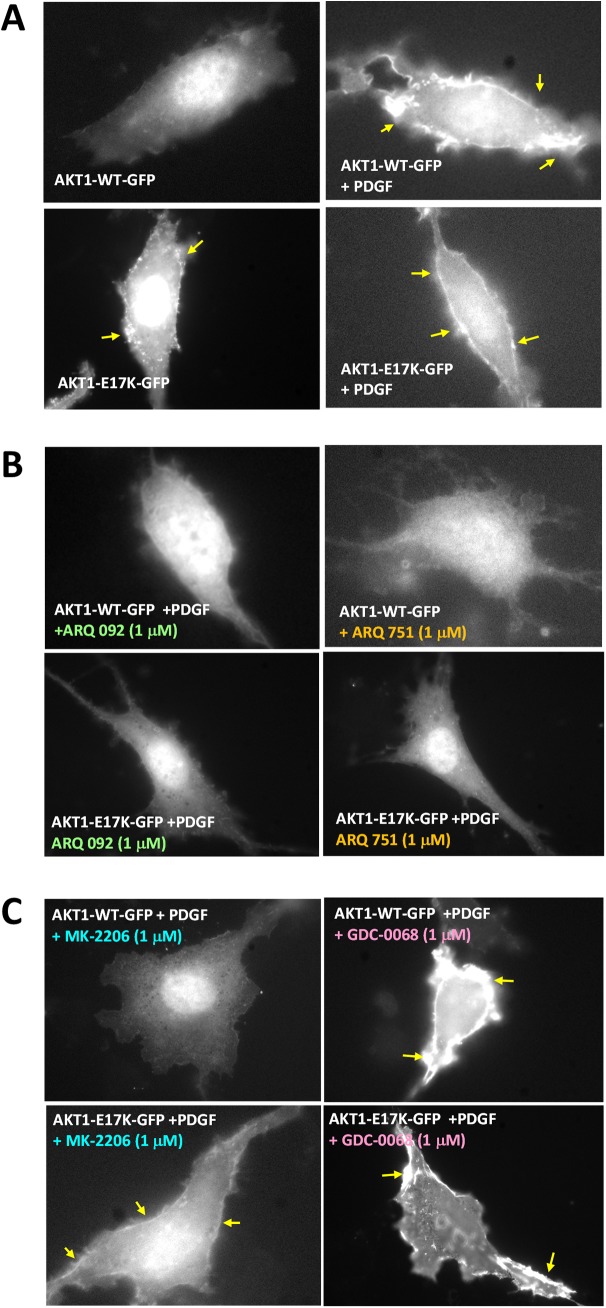
Plasma membrane translocation experiments conducted in AKT1-WT and E17K mutant forms of AKT1. NIH 3T3 cells were transiently transfected with either pcDNAAKT-WT-GFP or pcDNA-E17K-GFP, starved for 24 hours and then were treated with (A) DMSO (B) ARQ 092 or ARQ 751 or (C) MK-2206 or GDC-0068 at 1 μM for 2 hours. After cells were stimulated with PDGF-BB at 50 ng/ml for 10 minutes, membrane translocation of AKT1-WT and AKT1-E17K was detected by a fluorescent microscope.

To further investigate the nature of the inhibition of AKT1-WT and AKT1-E17K with AKT inhibitors and identify potential differences, plasma membrane translocation experiments were conducted with ARQ 092 and ARQ 751 as well as the reference compounds MK-2206 (allosteric AKT inhibitor) and GDC-0068 (ATP competitive AKT inhibitor). As shown in Fig 2A, AKT1-WT was localized to the plasma membrane only when stimulated with growth factor (PDGF-BB). However, oncogenic AKT1-E17K is constitutively localized to the membrane and growth factor augmented the localization. ARQ 092 and ARQ 751 both inhibit the first pivotal step in the activation of AKT, plasma membrane translocation of AKT-WT and AKT1-E17K irrespective of the presence of growth factors, and thus inhibited activation of AKT (Fig 2B). This suggests that these compounds can be active both when the pathway is stimulated (due either to growth factors or PI3K mutation) or when AKT is constitutively activated via a mutation. Both MK-2206 and GDC-0068 behaved as expected (Fig 2C). MK-2206 inhibited the plasma membrane translocation of AKT1-WT, but only weakly inhibited the membrane translocation of AKT1-E17K. GDC-0068 stimulated translocation of AKT1 and AKT1-E17K to the plasma membrane and showed the expected hyperphosphorylation of AKT1 [39].

### ARQ 092 and ARQ 751 potently and selectively inhibit AKT

Previously we reported the identification and pharmacological characterization of a novel series of allosteric AKT inhibitors [40]. The lead compound of these series showed excellent kinase selectivity and *in vivo* inhibition of AKT activation. The core moiety of the lead compound, (3-phenyl-3H-imidazo[4,5-b]-pyridin-2-yl)pyridin-2-amine was further optimized into the more potent ARQ 092 molecule. More recently, our next generation of AKT inhibitors including ARQ 751, were similarly optimized. We first evaluated the effect of ARQ 092 and ARQ 751 on AKT activity by performing *in vitro* biochemical assays using inactive full-length AKT1, 2 and 3. The full-length AKT rather than constructs containing only the AKT kinase domain was essential for potent biochemical inhibition by ARQ 092 [40]. ARQ 092 inhibited AKT1, 2, and 3 activities with IC_50_ values of 5.0, 4.5, and 16 nM, respectively, whereas ARQ 751 had IC_50_ values of 0.55, 0.81, and 1.3 nM, respectively. In comparison, MK-2206 had lower potency (40.5, 29.5 and 36.4 nM) against all three AKTs while GDC-0068 showed equivalent potency to ARQ 092 against AKT1 (Table 1). Selectivity profiling against a panel of 303 kinases was performed and showed that only six kinases (MARK1, 3 and 4, DYRK2, IRAK1, and Haspin) exhibited more than 50% inhibition by ARQ 092 at a concentration of 5 μM. The IC_50_ values of ARQ 092 for these six kinases were 129, 173, 180, 386, 806, and 1160 nM, respectively (Table 2). Notably, ARQ 751 (out of a panel of 245 kinases) did not inhibit any kinase other than full-length AKT1 by more than 50% at a concentration of 5 μM (Table 2). For the selectivity assays, full-length AKT2 and AKT3 were not available. However, full-length AKT 2 and AKT 3 were used in the biochemical assays discussed above further demonstrating that the full-length AKT is required for ARQ 092 or ARQ 751 to exert their inhibitory effect.

**Table 1:** ARQ 092 and ARQ 751 potency.

		Biochemical IC_50_ (nM)		
	Type	AKT1	AKT2	AKT3
ARQ 092	Allosteric	5.0	4.5	16
ARQ 751	Allosteric	0.55	0.81	1.3
MK-2206	Allosteric	40.5	29.5	36.4
GDC-0068	ATP-competitive	2.0	27.0	6.3

The biochemical IC_50_ values for ARQ 092, ARQ 751, MK-2206, and GDC-0068 were determined against full-length active form of AKT1, 2, and 3.

**Table 2:** ARQ 092 and ARQ 751 kinase selectivity against AKT-WT.

ARQ 092		ARQ 751	
Kinase	IC_50_ (nM)	Kinase	% Inhibition at 5 μM
MARK4	129	AKT1-FL	57
MARK3	173	Blk(h)	40
MARK1	180	Tie2	33
DYRK1	386	Haspin	30
IRAK1	806	Met	28
Haspin	1160	SGK3(119-end)	28
		PASK	26
		Pim-3	24
		LKB1	23
		CDK2/cyclinE	21
		ErbB4(HER4)	21
		Syk	21
		FAK	20
		Other 232 kinases	<20

The biochemical IC_50_ values of ARQ 092 against 303 kinases were determined (Carna Biosciences). The percent kinase inhibition of ARQ 751 was determined at a concentration of 5μM against a panel of 245 kinases.

### ARQ 092 and ARQ 751 inhibit AKT downstream signaling

**Fig 3:**
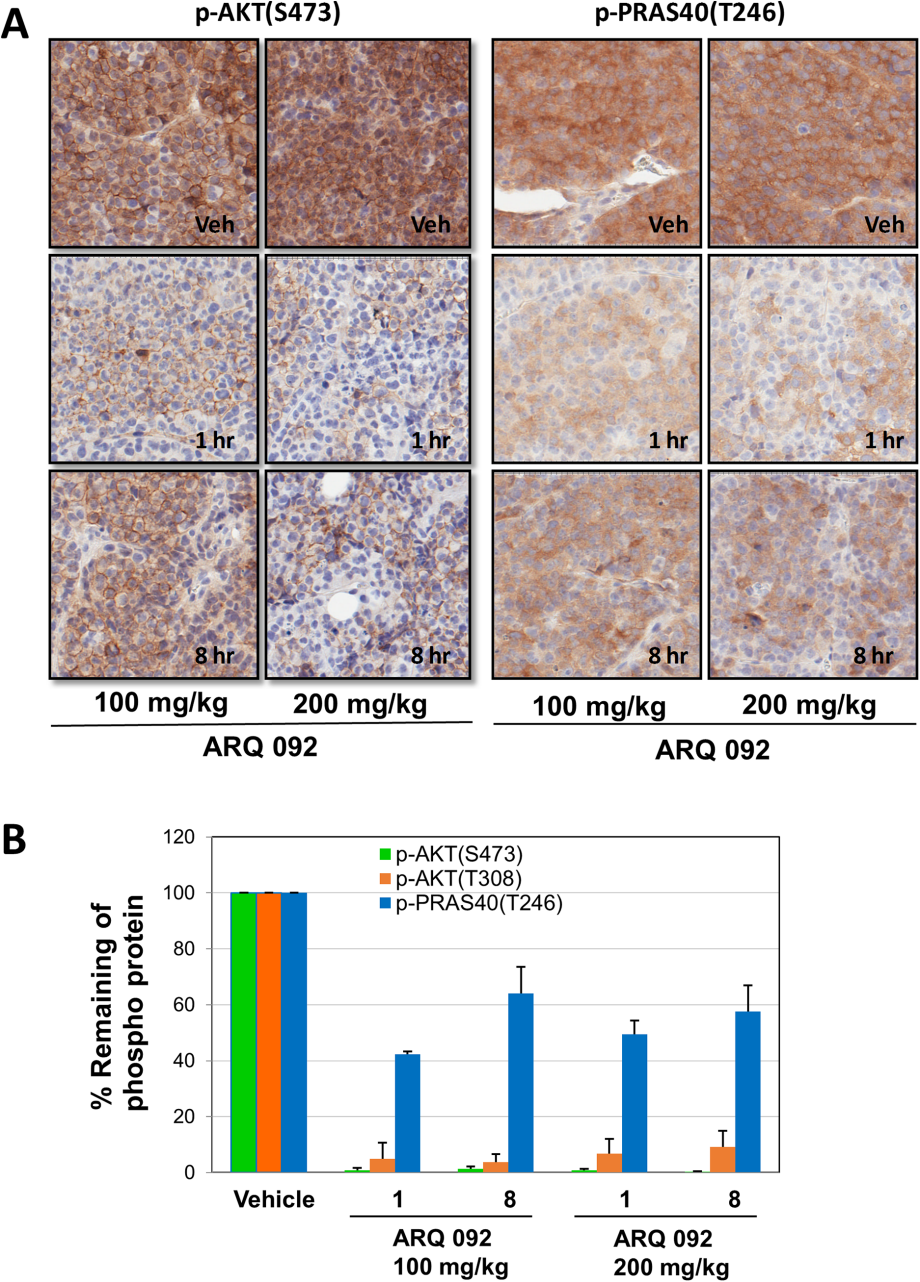
ARQ 092 inhibits AKT signaling in AN3CA mouse xenografts, *In Vivo*. (A) Tumor samples were assessed by IHC for p-AKT(S473) and p-PRAS40(T246). Veh: Vehicle. (B) p-AKT(S473) and (T308), and p-PRAS40(T246) were assessed by western blot analysis from tumor tissues from and AN3CA mouse xenograft after treatment with 100 or 200 mg/kg. The percentage remaining of the phosphorylated proteins is shown and the vehicle group was designated as 100%.

Next, we examined the effect of ARQ 092 and ARQ 751 on AKT phosphorylation at the threonine 308 and serine 473 amino acid residues as well as its substrate PRAS40 at threonine 246 in AN3CA endometrial cancer cells. ARQ 092 inhibited phosphorylation of AKT and PRAS40 in a concentration-dependent fashion with IC_50_ values of 62 nM for pAKT(T308), 40 nM for pAKT(S473) and 310 nM for pPRAS40(T246), demonstrating that ARQ 092 is able to inhibit AKT signaling (S3 Fig). ARQ 751 more potently inhibited phosphorylation of AKT and PRAS40 in a concentration-dependent fashion with IC_50_ values of 5 nM for pAKT(T308), 10 nM for pAKT(S473) and 49 nM for pPRAS40(T246) (S1 Table). ARQ 092 was also shown to inhibit phosphorylation of other downstream kinases in the pathway (e.g. pS6 and pGSK3β) in AN3CA cell lines (S4 Fig). Both ARQ 092 and ARQ 751 were found to inhibit the AKT signaling pathway *in vivo* (Figs 3, S5 and S6).

**Fig 4:**
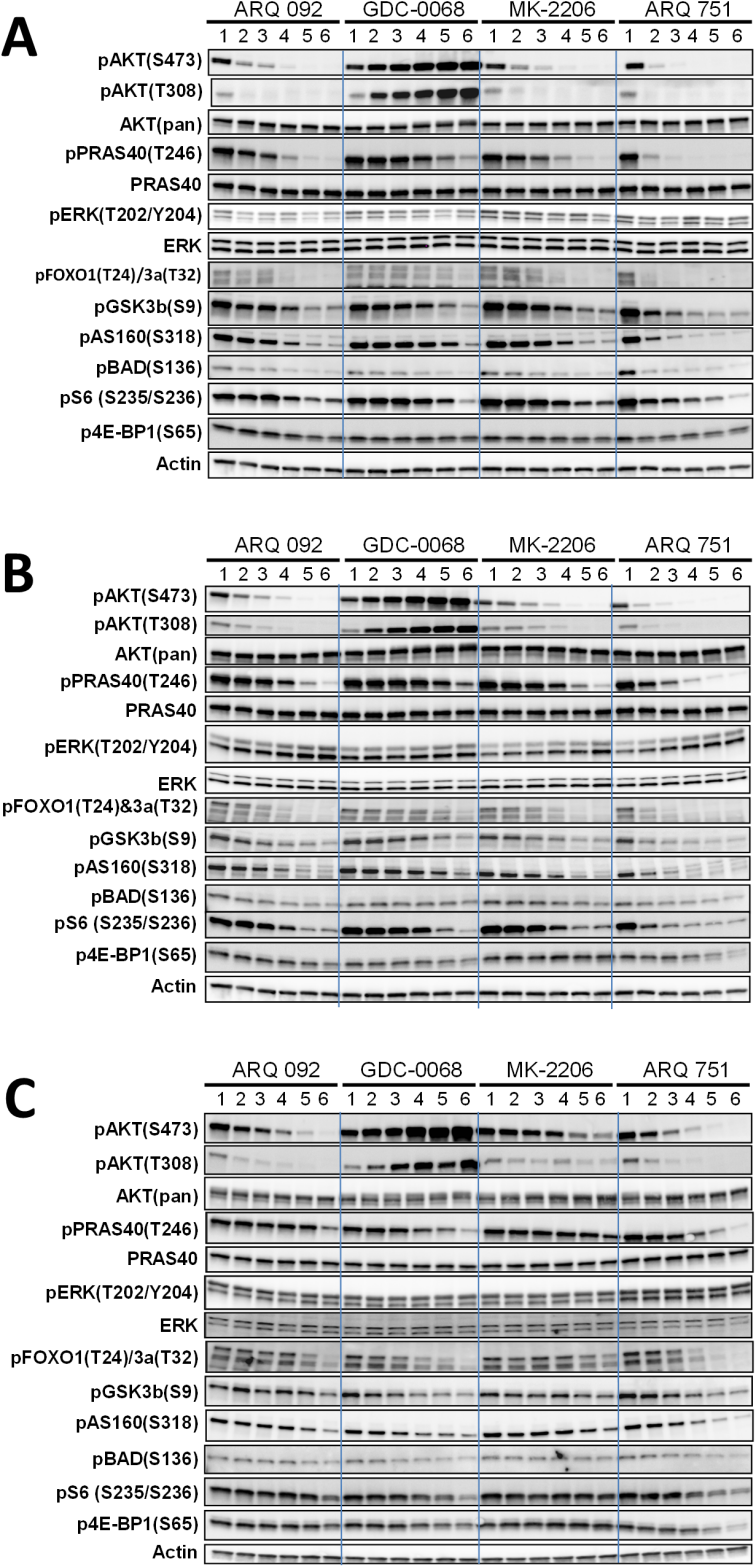
Comparison of AKT pathway inhibition by ARQ 092, ARQ 751, MK-2206, and GDC-0068 in MDA-MB-453, NCI-H1650, and KU-19-19 cells. (A) MDA-MB 453 breast cancer cell line (PIK3CAH1047R; Her2 amp), (B) NCI-H1650 NSCLC cell line (PTEN null), and (C) KU-19-19 bladder cancer cell line (AKT1-E17K&E49K; NRas Q61R) were treated with various concentrations (1 = 0, 2 = 0.012, 3 = 0.037, 4 = 0.11, 5 = 0.33, and 6 = 1 μM) of ARQ 092, ARQ 751, MK-2206 or GDC-0068 for 2 hours. pAKT(S473), pAKT(T308), pPRAS40(T246), pFOXO1(T24) /3a(T36), pGSK3β(S9), pAS160(S318), pBAD(S136), pS6(S235/236) and p4E-BP1(S65) and phospho ERK were assessed by western blot analysis.

To further determine the effect of ARQ 092 and ARQ 751 on the AKT pathway, we selected three cell lines with an activated AKT pathway, MDA-MB 453(PIK3CAH1047R), NCI-H1650 (PTEN null) and KU-19-19 (AKT-E17K, AKT-E17K + E49K, NRasQ61R). As shown in Fig 4, all AKT inhibitors tested in this study suppressed AKT downstream biomarkers but not pERK. ARQ 092 and ARQ 751 showed a dose-dependent effect not only on AKT direct substrates including PRAS40, GSK3β, FOXO, BAD, and AS160 but also on the AKT-dependent activity of mTORC1, which has been shown to modulate changes in S6 and e4BP1. The most significant difference in potency was observed in the activating AKT mutant KU-19-19 cells carrying AKT1-E17K + E49K. The KU-19-19 cells contain three transcripts: AKT1-WT, AKT1-E17K, and AKT1-E17K + E49K [31, 41]. To determine whether the AKT inhibitors tested in this study exert anti-proliferative activity, we performed cell proliferation assays. Only the MDA-MB-453 cells were responsive to AKT inhibitors. ARQ 092 showed higher anti-proliferative activity than MK-2206 or GDC-0068 while ARQ 751 was the most potent of the four compounds. The IC_50_ value of ARQ 751 in MDA-MB-453 cells was about 12-fold lower than ARQ 092, 26 fold lower than MK-2206, and 100-fold lower than GDC-0068 (S2 Table). Overall, the inhibitory effect of the allosteric AKT inhibitors ARQ 092 and ARQ 751 on the AKT pathway was greater in MDA-MB-453 and NCI-H1650 cells than that observed in KU-19-19 cells. ARQ 751 strongly inhibited the phosphorylation of AKT1-E17K and its downstream markers. Therefore, despite being an allosteric AKT inhibitor, ARQ 751 possesses an inhibitory potency similar to that of an ATP-competitive inhibitor against AKT1-E17K and AKT1-E17K + E49K mutants.

### ARQ 092 and ARQ 751 exhibit strong anti-proliferative activity in PIK3CA mutant cell lines

**Fig 5:**
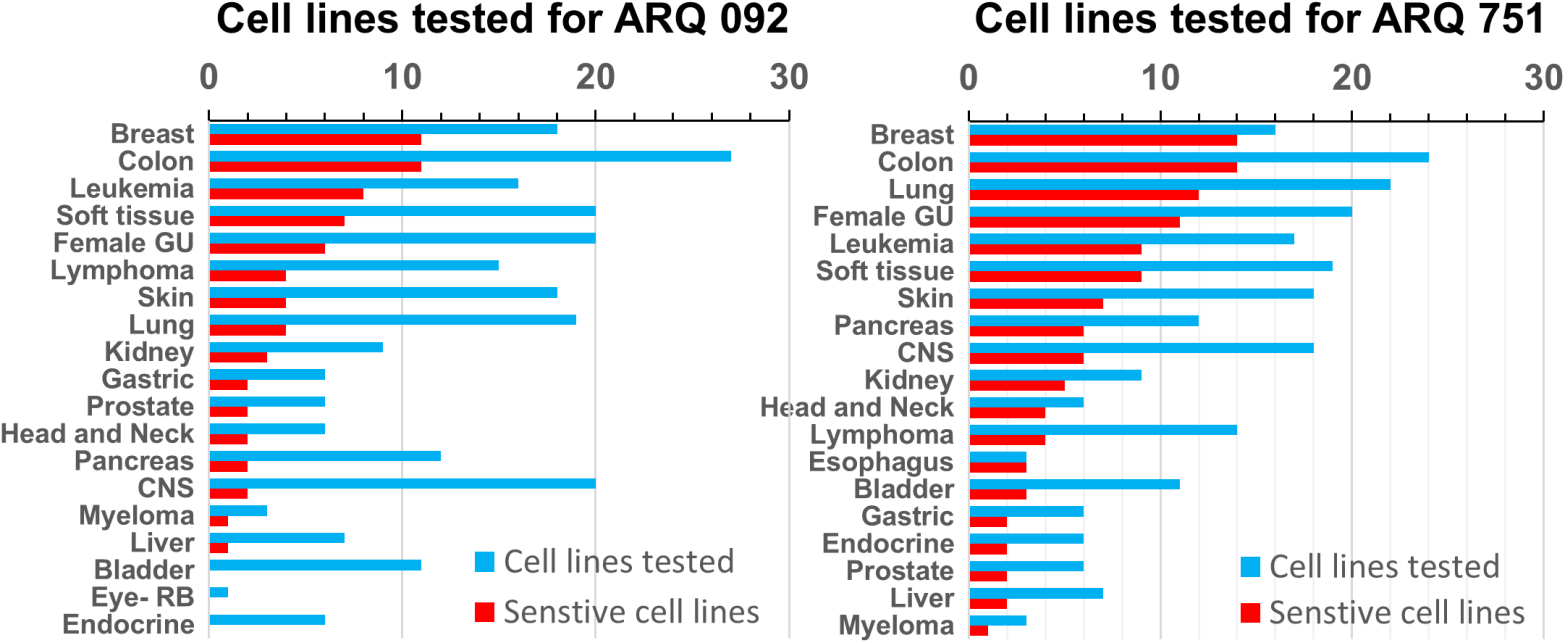
Determination of the sensitivity of ARQ 092 and ARQ 751 with different cancer cell types. Plots of cancer type versus drug sensitivity and number of cell lines per cancer type were prepared for ARQ 092 (left panel) and ARQ 751 (right panel).

To determine the anti-proliferative effect of ARQ 092 and ARQ 751 in cancer cell lines, the GI_50_ for a panel of 240 cell lines from the OncoPanel screen was determined. ARQ 092 and ARQ 751 both displayed a range of potencies against a spectrum of different cancer cell types (Fig 5). Breast, leukemia and colorectal cancer cells were the most sensitive (GI_50_ < 1 μM) to ARQ 092 with sensitivity rates of 61% (11 out of 18 cell lines), 50% (8 out of 16 cell lines) and 41% (11 out of 27 cell lines), respectively. Esophageal, breast, and head and neck cancer cells were the most sensitive (GI_50_ < 1 μM) to ARQ 751 with sensitivity rates of 100% (3 out of 3 cell lines), 87.5% (14 out of 16 cell lines) and 67% (4 out of 6 cell lines), respectively. For leukemia and colorectal cancer cells, ARQ 751 had sensitivity rates of 53% (9 out of 17 cell lines) and 56% (14 out of 25 cell lines), respectively.

**Fig 6:**
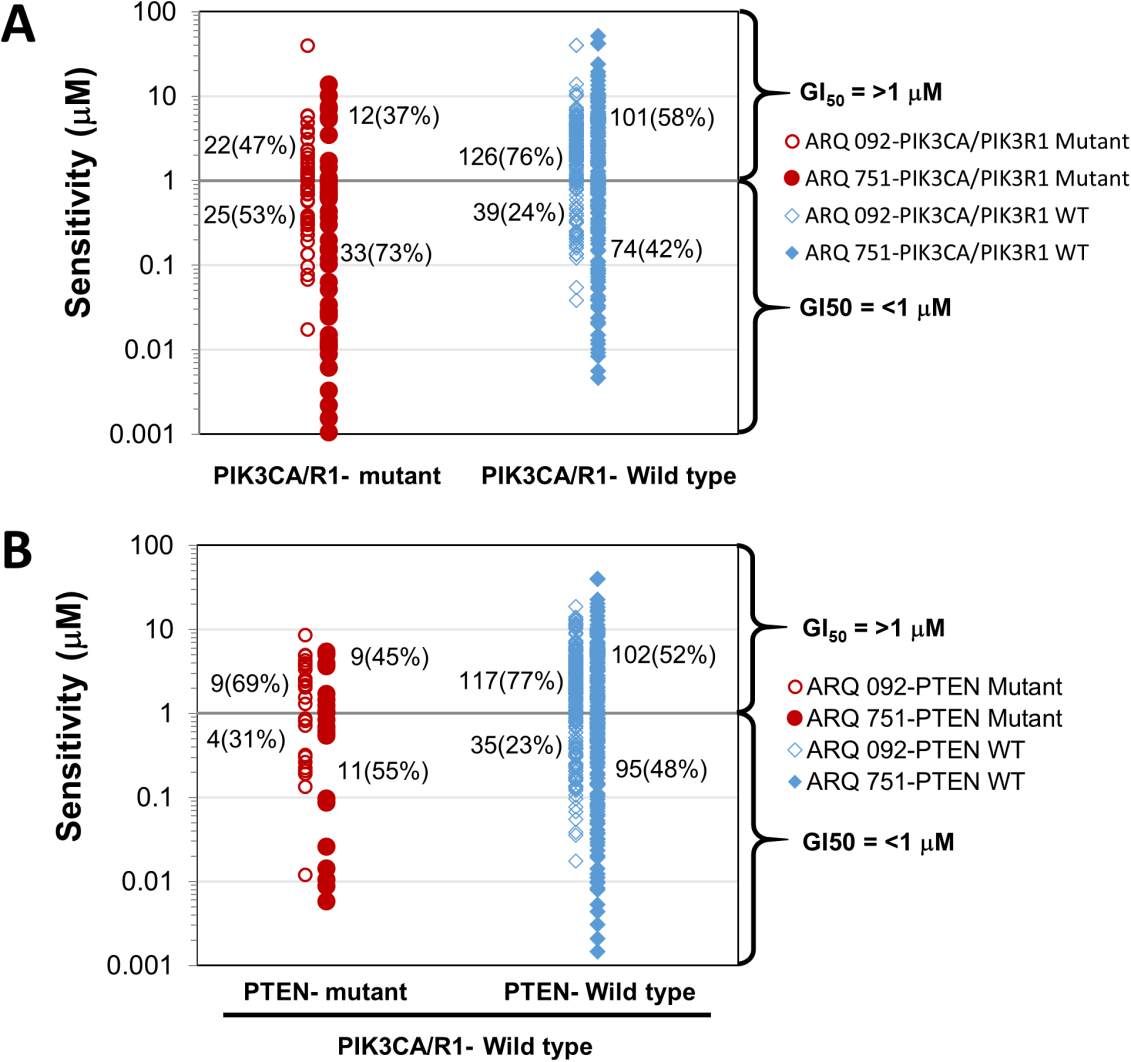
PIK3CA/PIK3R1 mutational status is associated with increased ARQ 092 and ARQ 751 sensitivity. Somatic mutation analysis was performed for ARQ 092 and ARQ 751 sensitive (GI_50_<1 μM) and resistant (GI_50_ ≥1 μM) groups for 212 cancer cell lines. Twenty-eight of 240 cells were excluded due to unavailability of mutation status. (A) Scatter plot shows the correlation between ARQ 092 and ARQ 751sensitivity and PIK3CA/R1 mutations in comparison to WT-PIK3CA/PIK3R1. (B) Scatter plot shows the correlation between ARQ 092 and ARQ 751 sensitivity and PTEN mutations in comparison to WT-PTEN.

To investigate whether any somatic mutations correlated to ARQ 092 sensitivity, we divided cell lines into ARQ 092 sensitive (GI_P_<1 μM) or resistant (GI_50_≥1 μM) groups and analyzed their mutational status with respect to PIK3CA/PIK3R1 and PTEN mutations. This was accomplished using the Catalogue of Somatic Mutations in Cancer (COSMIC) database [42]. Of the 240 cell lines tested, 212 of them were assessed. This analysis showed there were 22% of cell lines (47 out of 212 cell lines) with PIK3CA/PIK3R1 mutations, 10% (22 out of 212 cell lines) with PTEN mutations, and 4.2% (9 out of 212 cell lines) with both mutations. As depicted, in Fig 6A, approximately 53% (25 out of 47) of cell lines with PIK3CA/PIK3R1 mutations were sensitive to ARQ 092 (GI_50_<1 μM). In comparison, only 24% (39 out of 165) of the cell lines with WT-PIK3CA/PIK3R1 (p = 0.0002) were sensitive. For ARQ 751, approximately 73% (33 out of 45) of cell lines with PIK3CA/PIK3R1 mutations were sensitive to ARQ 751 (GI_50_<1 μM). In comparison, only 42% (74 out of 175) of the cell lines with WT-PIK3CA/PIK3R1 (p = 0.0002) were sensitive. Interestingly, PTEN status (Fig 6B) did not appear to have predictive value since 31% of the mutant PTEN cell lines (4 out of 13 cell lines) and 23% of the WT-PTEN cell lines (35 out of 152 cell lines) among WT-PIK3CA/PIK3R1 cell lines exhibited a similar sensitivity to ARQ 092 (p = 0.508). To further evaluate the predictive value of PIK3CA/PIK3R1 mutations, we examined the potential correlation of ARQ 092 and ARQ 751 sensitivity with PIK3CA mutations in breast cancer cell lines and found 88% and 100% (7 out of 8 or 8 out of 8 cell lines) of breast cancer cell lines with PIK3CA mutations were sensitive to ARQ 092 or ARQ 751, respectively (S3A and S3B Table). Additionally, the data showed that breast cancer cells expressing hormone receptors and/or overexpressing HER2 were sensitive to ARQ 092 or ARQ 751.

### Xenograft Models and endometrial PDX models demonstrate that ARQ 092 and ARQ 751 are highly potent in several cancer types including those harboring AKT1-E17K mutations

**Fig 7:**
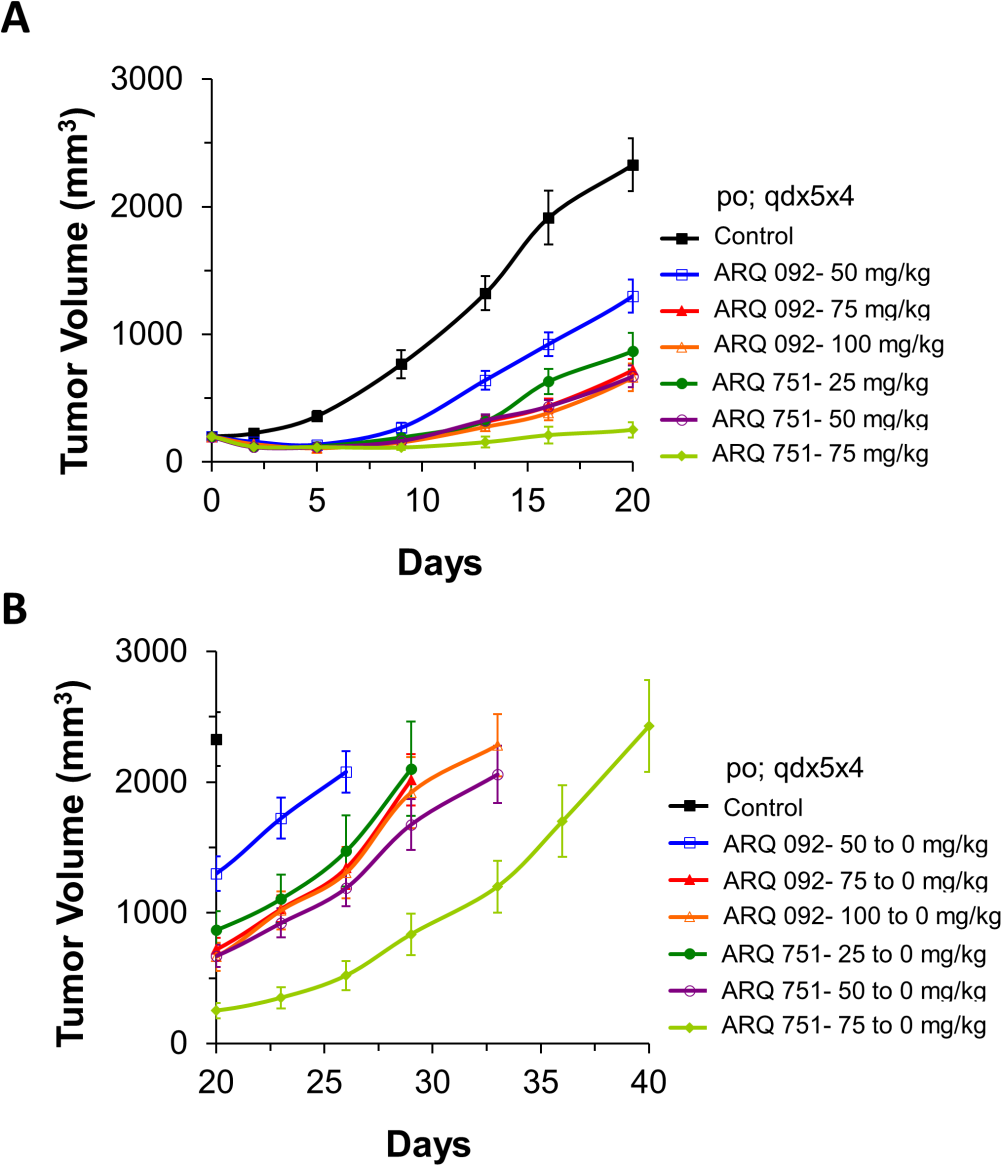
Antitumor activity in endometrial PDX mouse xenograft models after treatment with ARQ 092 or ARQ 751. An endometrial PDX mouse xenograft model, ARQ 092 dosed at 50, 75 or 100 mg/kg or ARQ 751 dosed at 25, 50, 75 mg/kg in a schedule of 5 days on and 2 days off for 20 days. (A) Plot of tumor growth inhibition. (B) Plot of tumor regrowth after removal of drug. (Day 20 to Day 40).

To demonstrate that ARQ 092 and ARQ 751 are effective against AKT1-E17K mutations *in vivo* we tested them in PDX models from an endometrial tumor harboring the AKT1-E17K mutant and several other major mutations (ARID1A, BRCA1/2, TP53, APC). In this study, mice were dosed at 50, 75, and 100 mg/kg with ARQ 092 and 25, 50 and 75 mg/kg with ARQ 751 on a dosing schedule of 5 days dosing followed by a 4 day dosing holiday for 20 days. As shown in Fig 7A, both ARQ 092 at 50, 75, and 100 mg/kg and ARQ 751 at 25, 50 and 75 mg/kg showed potent tumor growth inhibition of 48, 75 and 78% and 68, 78 and 98%, respectively. Consistent with the *in vitro* data, these results confirmed that both ARQ 092 and ARQ 751 inhibited AKT1-E17K *in vivo*. Tumor regrowth analysis showed that significant tumor growth (after removal of drug) was delayed for at least two weeks compared to the control group for all dose levels of ARQ 751 and at 75 and 100 mg/kg dose levels for ARQ 092 (Fig 7B).

The effect of ARQ 092 in endometrial cancer was further evaluated endometrial PDX models. Based on the overall panel screen at the maximum tolerated dose (MTD) of 100 mg/kg among twenty-three endometrial PDX models tested, ARQ 092 was most active towards ST189, ST1529, ST1619, ST259 and ST993 models showing over 90% tumor growth inhibition (S4 Table). Genomic sequence analysis of PDX models revealed ARQ 092 sensitive PDX models are heavily altered with PI3K pathway genes, harboring AKT1, AKT2, PIK3CA and TSC1/2 mutations and PTEN loss. This preliminary efficacy screening study indicated that ARQ 092 was highly active in a subset of endometrial tumors that harbor PI3K pathway gene mutations.

**Fig 8:**
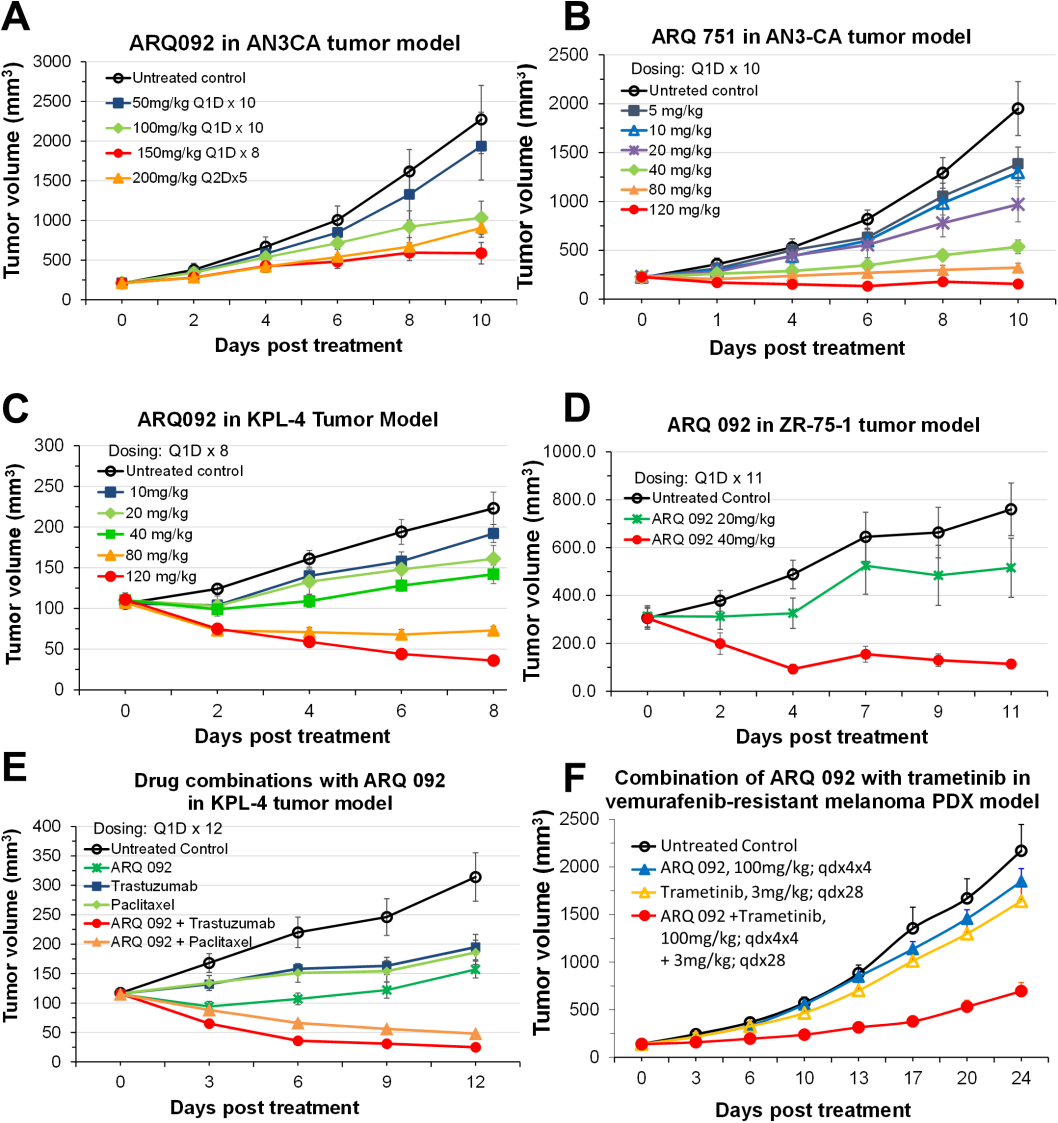
Antitumor activity in various mouse xenograft models after treatment with ARQ 092 or ARQ 751. (A) AN3CA xenograft model treated with ARQ 092, (B) AN3CA xenograft model treated with ARQ 751, (C) KPL-4 xenograft model treated with ARQ 092, (D) ZR-75-1 xenograft model treated with ARQ 092, (E) KPL-4 xenograft model treated with ARQ 092 as a single agent or combined with trastuzumab or paclitaxel, (F) A PDX melanoma model (ST052C) that contained Braf V600E and PIK3CA (H1047R) mutations treated with ARQ 092 as a single agent or combined with trametinib.

Additionally, we assessed the anti-tumor activity of ARQ 092 in *in vivo* mouse models implanted with AN3CA cells (mutant PIK3R1, PTEN deficient, and activating mutation of FGFR2) [43], KPL-4 cells (over-expression of HER2 and mutation in PIK3CA) [44]; and ZR-75-1 cells (ER positive, PTEN deficient) [45, 46]. ARQ 751 was assessed only in AN3CA mouse xenograft models. Administration of ARQ 092 at 100 mg/kg suppressed AN3CA tumor growth by about 55% after daily dosing for 10 days, whereas only a 15% reduction was observed in the 50 mg/kg group (Fig 8A). ARQ 092 dosed at 200 mg/kg every other day produced similar tumor growth inhibition (60% versus 74%) to the group receiving daily ARQ 092 at 100 mg/kg. At an oral dose of 100 mg/kg, ARQ 092 reached C_max_ plasma concentrations of ~2 μM (S7A Fig). When ARQ 751 was dosed QD for ten days at 5, 10, 20, 40, 80, and 120 mg/kg, it resulted in tumor growth inhibition of 29, 33 (p<0.05), 50 (p<0.001), 73 (p<0.001), 83 (p<0.001), and 92% (p<0.001), respectively, in AN3CA mouse xenograft models (Fig 8B). At oral dose levels ≥20 mg/kg, ARQ 751 reached C_max_ plasma concentrations of ≥2 μM (S7B Fig). Similar tumor growth inhibition by ARQ 092 was observed in both the KPL-4 (TGI = 84%, p<0.001, 120 mg/kg) and ZR-75-1 (TGI = 85%, p<0.001, 40 mg/kg) breast cancer mouse xenograft models (Fig 8C and 8D). The sensitivity of ARQ 092 in ZR-75-1 tumors could be due to the combined activation of PI3K by the PTEN mutation and moderate expression of HER2. Previous studies have shown that ZR-75-1 cells are responsive to PI3K/AKT/mTOR inhibitors [47]. Taken together, these data demonstrate that ARQ 092 is effective in suppressing tumor growth *in vivo*. Additionally, ARQ 092 and ARQ 751 were generally well-tolerated at dose levels up to 120 mg/kg and no ARQ 092 or ARQ 751 related animal deaths were observed in any of the *in vivo* studies. Some animals experienced weight loss (generally limited to ≤10%) at dose levels of ARQ 092 or ARQ 751 ≥100 mg/kg.

A number of studies have shown that intrinsic or acquired activation of the PI3K/AKT/mTOR pathway results in resistance to targeted therapy and chemotherapy [48]. HER2-positive breast cancer with activating mutations of PIK3CA do not respond to trastuzumab treatment [49]. Administration of ARQ 092, trastuzumab, or paclitaxel as single agents, inhibited tumor growth by 50%, 38% and 41%, respectively, in comparison to the untreated control group (Fig 8E). The combination of ARQ 092 with trastuzumab or paclitaxel induced marked tumor suppression in comparison to the effect of each single agent alone, showing 92% (trastuzumab) or 85% (paclitaxel) growth inhibition of KPL-4 tumors (p<0.001), respectively. These data indicate that ARQ 092 enhances the effect of trastuzumab or paclitaxel in an *in vivo* breast cancer model compared to single agent therapy. Our findings are consistent with the results published for similar studies [46, 47, 50]. Lastly, Fig 8F shows the results of study conducted in an acquired vemurafenib-resistant PDX melanoma model which contained Braf(E600V) and PIK3CA(H1047K) mutations. The combination of ARQ 092 and trametinib exhibited 73% TGI (p<0.05), whereas ARQ 092 and trametinib as single agents only had limited effect (16% and 26% respectively, p<0.05).

## Discussion

We have presented data demonstrating that ARQ 092 and the next generation compound ARQ 751 are potent and highly selective allosteric pan AKT inhibitors. Given the recent attention AKT1-E17K mutations has received, targeting this mutation in cancer and other diseases might serve as a potential niche for potent and selective AKT inhibitors such as ARQ 092 and ARQ 751. With that in mind, we tested the ability of both ARQ 092 and ARQ 751 to bind to and inhibit AKT1-E17K1. Biochemical *in vitro* binding assays clearly demonstrated that ARQ 092 and ARQ 751 bind tightly to both the AKT1-WT and the AKT1-E17K mutant at nanomolar concentrations. Previously, we reported the crystal structure of an analogue of ARQ 092 bound to the allosteric pocket formed between the kinase and PH domain interface [39]. We also showed that the extensive interdomain interactions play a critical role in stabilizing AKT in the autoinhibited state for inhibitor interaction. Therefore, it can be postulated that the strong binding of ARQ 092 and ARQ 751 to both the WT and mutant forms of AKT could occur as a result of the better stabilization of the kinase and PH domain interface. The oncogenic E17K mutation on the PH domain of AKT weakens the kinase and PH domain interface leading to enhanced membrane localization and activation of kinase function of AKT [24].

**Fig 9:**
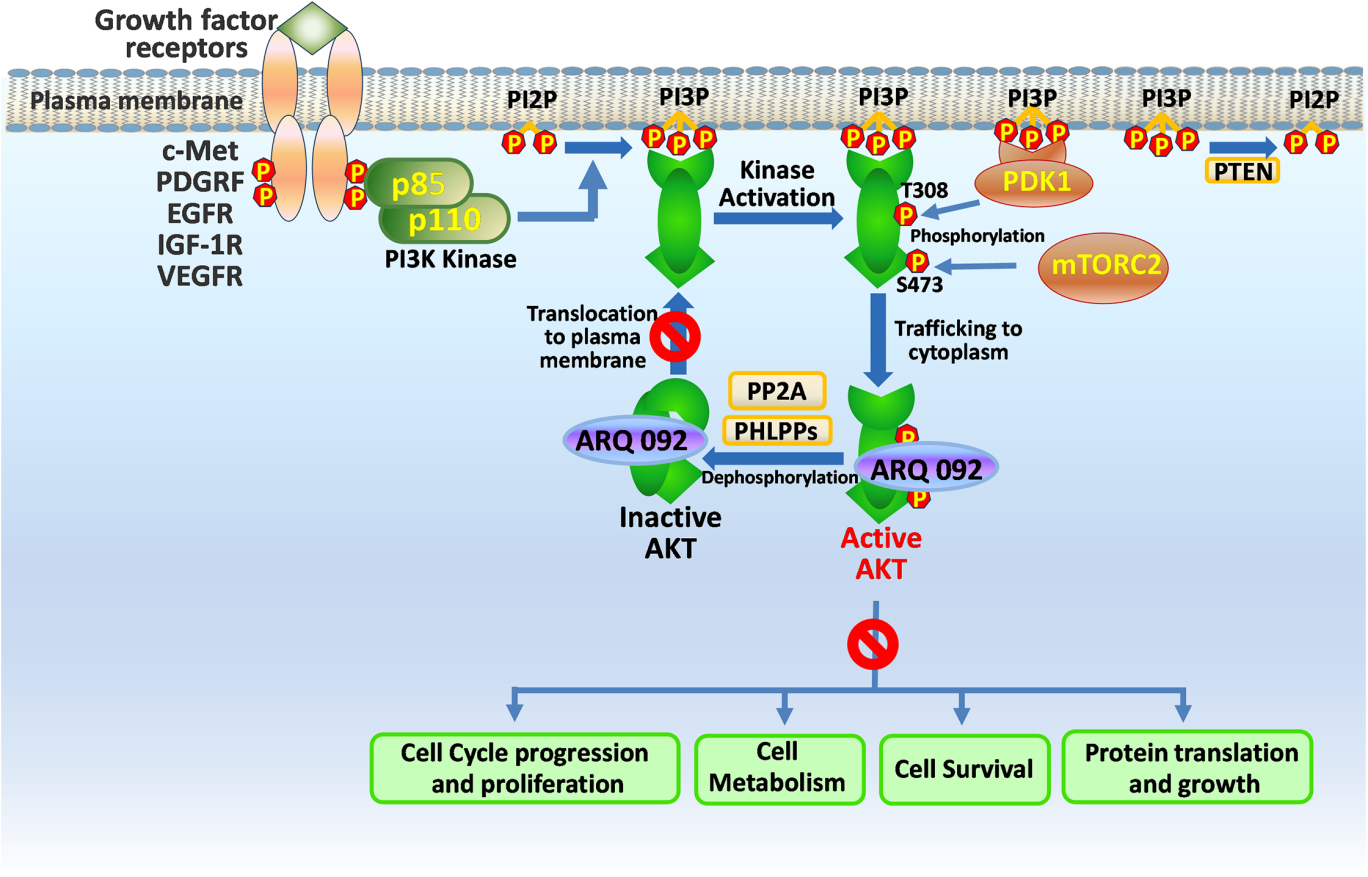
Proposed AKT pathway inhibition by ARQ 092 (and ARQ 751).

Because the E17K mutation activates AKT1 via localization to the plasma membrane, we next studied the ability of ARQ 092 and ARQ 751 to disrupt this process. The results of these experiments showed that ARQ 092 and ARQ 751 both inhibited the first pivotal step in the activation of AKT (plasma membrane translocation of AKT-WT and AKT1-E17K) irrespective of the presence of growth factors and thus inhibited PI3K dependent activation of the protein. The proposed pathway inhibition by ARQ 092 (and ARQ 751) is presented in Fig 9. Because allosteric inhibitors (such as ARQ 092 and ARQ 751) bind to both the active and inactive forms of AKT, they appear to not only suppress AKT activation of the active form (as do ATP-competitive inhibitors) but also suppress AKT activation of the inactive form by disrupting membrane translocation.

In cellular assays, both ARQ 092 and/or ARQ 751 potently inhibited elevated AKT activity and showed pathway knockdown in cancer cell lines driven by PIK3CAH1047R, PTEN null, and AKT1-E17K mutations. ARQ 092 was also shown to be active in cells derived from patients with PS which is driven by an AKT1-E17K mutation [51]. Most significantly, in an *in vivo* study, ARQ 092 and ARQ 751 showed strong anti-tumor activity against a homologous AKT1 mutant (AKT1-E17K) endometrial cancer patient-derived xenograft (PDX) model. The results of this study clearly showed that both ARQ 092 and ARQ 751 not only potently inhibited tumor growth in this E17K mutant model, but also significantly delayed tumor regrowth post treatment. Additionally, we showed that ARQ 092 had strong tumor growth inhibition not only as a single agent, but also in combination with other drugs in a number of cancer tumor models. Interestingly, inhibition of AKT activity by ARQ 092 reversed vemurafenib-resistance induced by the activating mutation of PIK3CA when combined with MEK inhibitor.

Both ARQ 092 and ARQ 751 were evaluated against AKT-WT as well as against PIK3CA activating and PTEN loss mutations of AKT. This analysis showed 88% and 100% of breast cancer cell lines with PIK3CA mutations and a GI_50_ < 1μM were sensitive to ARQ 092 or ARQ 751, respectively. The data also showed that breast cancer cells (triple negative breast excluded) expressing hormone receptors and/or overexpressing HER2 were sensitive to ARQ 092 or ARQ 751. Overall, based on currently published data, ARQ 092 and/or ARQ 751 are generally more potent in breast cancer cell lines than most other AKT inhibitors (S8 Fig). Further, kinase profiling showed that both ARQ 092 and ARQ 751 are very selective pan-AKT inhibitors. Such high selectivity may distinguish ARQ 092 and ARQ 751 from ATP-competitive inhibitors by providing not only enhanced efficacy but also better predictability of toxicities associated only with inhibition of AKT and not of additional kinases as is often observed with less selective inhibitors [52].

In a retrospective analysis, Janku et al. reported that patients with tumors harboring PIK3CA mutations, particularly the hotspot mutation H1047R, had a favorable clinical outcome when treated with PI3K/AKT/mTOR pathway inhibitors [53]. Our results divulged that breast cancer, leukemia and colorectal carcinomas were the most sensitive cancer cell types to ARQ 092 and that cell lines bearing PIK3CA/PIK3R1 mutations were more responsive to ARQ 092 (53%) compared to cells containing WT-PIK3CA/PIK3R1 (24%). No significant difference in sensitivity to ARQ 092 was observed between cell lines with PTEN mutations (31%) compared to cell lines with WT-PTEN (23%). These findings suggest that PIK3CA/PIK3R1, but not PTEN mutations are predictive of ARQ 092 sensitivity. Our observations were confirmed in breast cancer cell lines as the majority of them with PIK3CA mutations were shown to be sensitive to ARQ 092 and ARQ 751.

The importance of PIK3CA mutation versus PTEN loss in relation to clinical benefit from treatment with PI3K/AKT/mTOR inhibitors remains ambiguous. In some cases, depending on cancer cell type, PIK3CA mutation appears to show positive correlation, but PTEN loss does not. For instance, Weigelt et al. have shown that PIK3CA, but not PTEN correlates with the sensitivity of breast cancer cells to the mTOR inhibitor everolimus [54, 55]. However, in another study with a panel of human cancer cell lines derived from various tissues, the sensitivity was found to correlate with both PIK3CA and PTEN loss [56]. Similarly, both PIK3CA mutation and PTEN loss correlated with MK-2206 sensitivity in breast cancer cell lines [57]. In a recent study from patient-derived tumorgrafts of head and neck cancers, Liu et al. revealed that PIK3CA or PIK3R1 mutations could predict sensitivity to the PI3K/mTOR inhibitor BEZ-235 [58]. Although some researchers have shown that hotspot mutations such as H1047R or E545K may predict response to PI3K/AKT/mTOR inhibitors [59], we did not see a similar correlation with our compounds in the cell lines we tested (results not reported). Perhaps one explanation for this lack of consistency lies with the genomic heterogeneity of primary tumors which presents a very complex scenario in defining predictive biomarkers for PI3K/AKT/mTOR inhibitors. Consistent with this hypothesis, Hanrahan et al. have reported that the genetic and functional analysis of ovarian cancer cell lines and tumors, mutational status of PIK3CA or PTEN alone may not be sufficient in predicting sensitivity to AKT inhibitors [60]. Overall, the results from these studies suggest that not only the presence of PIK3CA mutation, but also the specific type of cancer, may be important considerations in successfully targeting this mutation. Hence, the key components influencing response to AKT inhibitors, whether related to a specific cancer type, genetic feature, protein expression and/or AKT enzyme activity need to be further investigated [11, 60, 61]. Nonetheless, we have shown that somatic mutations of PIK3CA or PIK3R1 predicted ARQ 092 and ARQ 751 response *in vitro*. We believe that information obtained from a comprehensive genetic patient tumor profile and AKT phosphorylation status could aid in predicting responsiveness to ARQ 092 and ARQ 751 in a clinical setting. Currently, ARQ 092 is being evaluated in patients harboring AKT1-E17K and PIK3CA mutations in phase 1 clinical studies. Consistent with our preclinical data, early clinical results showed partial responses in patients harboring AKT1 and PIK3CA mutations [62].

In summary, we have shown that two allosteric inhibitors (ARQ 092 and ARQ 751) potently inhibit AKT1-E17K mutants both *in vitro* and *in vivo*. The high potency and high selectivity of these compounds warrant further clinical investigation in patients with cancer (and other diseases such as PS) targeting not only AKT1-E17K but also the PI3K/AKT pathway activated by PIK3CA mutations.

## Supporting Information

S1 Fig Chemical structure of the core moiety of ARQ 092 and ARQ 751[40]. (TIF)

S2 Fig Effect of ARQ 092 and ARQ 751 on Phosphorylation of Endogenous WT AKT in 293T Cells. Endogenous WT AKT phosphorylation status was assessed in the same 293T cells transiently transfected with AKT-E17K-GFP. Both ARQ 092 and ARQ 751 inhibit pAKT.
(TIF)

S3 Fig ARQ 092 inhibits AKT activity in cells. AN3CA cells were treated with various concentrations of ARQ 092 for two hours and stimulated with 100 ng/ml EGF and 100 nM insulin for 15 minutes. P-AKT(T308) and (S473) and phosphorylation of its downstream substrate PRAS40 were assessed by western blot analysis. The IC_50_ was determined for p-AKT(T308) and (S473) and p-PRAS40(T246).
(TIF)

S4 Fig Effect of ARQ 092 on pAKT, pPRAS40, pS6, pGSK in AN3CA cells. AN3CA cells were treated with ARQ 092 at 0, 0.03, 0.1, 0.3, 1, and 3 uM for 2 hours (n = 1). pAKT(S473), pPRAS40(T246), pS6(S235/S236) and pGSK3a/b(S21/S9) were assessed using RPMA (Theranostics Health).
(TIF)

S5 Fig ARQ 092 inhibits AKT signaling in BT474 mouse xenografts, *in vivo*. A: p-AKT (S473) and (T308), and p-PRAS40(T246) were assessed by western blot analysis from tumor tissues from and BT474 mouse xenograft after treatment with ARQ 092 at 200 mg/kg. The percentage remaining of the phospho proteins is shown and the vehicle group was designated as 100%. B: The same tumor samples were assessed by IHC for p-AKT(S473) and p-PRAS40(T246). Veh: Vehicle.
(TIF)

S6 Fig ARQ 751 inhibits AKT signaling *in vivo* in AN3CA mouse xenografts. Inhibitory effect of ARQ 751 on AKT signaling pathway in AN3CA mouse xenografts at single dose levels of 5, 10, 20, 40, 80, and 120 mg/kg. Left panels show p-AKT and p-PRAS levels at 6 hours while the right panels show these levels at 24 hours. Note: A212-8153 = ARQ 751.
(TIF)

S7 Fig Graphs showing plasma concentration-time profiles of ARQ 092 and ARQ 751 in AN3CA xenograft mouseand. A: AN3CA xenograft mice were dosed iv at 5 mg/kg or PO with 100 mg/kg with ARQ 092. Blood samples were collected at 5, 15, and 30 minutes, and 1, 2, 4, and 8 hours post IV dose; 30 minutes, 1, 2, 4, 8, and 16 hours post PO dose. Blood samples were centrifuged and the plasma was collected. Plasma samples were analyzed by LC/MS/MS. Plasma concentration verse time profiles and PK parameters were determined following 5 mg/kg single IV and 100 mg/mg single PO ARQ 092 administration to mice. B: AN3CA xenograft mice were treated orally with ARQ 751 at dose levels of 5, 10, 20, 40, 80 and 120 mg/kg. Blood samples were collected at 0, 1, 2, 4, 6, 24 hours post-dose of ARQ 751 for measurement of the plasma concentrations. ARQ 751 plasma concentrations were determined using LC/MS/MS.
(TIF)

S8 Fig Plot showing comparison of sensitivity of all published AKT inhibitors in breast cell lines to ARQ 092 and ARQ 751. Comparison of the GI_50_ of the leading AKT inhibitors against a panel of breast cancer cell lines demonstrates that ARQ 092 is more potent or equally potent compared to leading AKT inhibitors while ARQ 751 is the most potent in 9 out of 13 breast cancer cell lines.
(TIF)

S1 Methods Supplemental Methods. (DOCX)

S1 Table Table of EC_50_ values of ARQ 751 for p-AKT and p-PRAS40 in AN3CA cells. The inhibitory activity of ARQ 751 on the Akt signaling pathway in the human endometrial cancer AN3 CA cell line using cell-based ELISA. The EC_50_ values for the inhibition of p-Akt (Thr308), p-Akt (Ser473), and p-PRAS40 (Thr246) were 5, 10, and 49 nM, respectively.
(DOCX)

S2 Table Table of ARQ 092 and ARQ 751 IC_50_ data in select cancer cell lines. Select cancer cell lines were treated with various concentrations of ARQ 092, ARQ 751, MK-2206, or GDC-0068 for single agent. IC_50_ was calculated using Activity Base. Data shown as mean ± standard deviation. MDA-MB-453: 3000 cells; NCI-H1650: 2000; and KU-19-19: 2500 cells.
(DOCX)

S3 Table Tables showing PIK3CA mutation and its association with increased ARQ 092 and ARQ 751 sensitivity in breast cancer cells. The correlation of PIK3CA/PIK3R1 mutation with A: ARQ 092 and B: ARQ 751 sensitivity in breast cancer cell lines was determined as shown on this table. Seven out of 8 (88%) cell lines bearing PIK3CA/PIK3R1 mutations are sensitive to ARQ 092 (GI_50_<1 μM). Among all breast cancer cell lines tested, 11 out of 18 (61%) cell lines are sensitive to ARQ 092 while triple negative breast cancer cell lines are resistant (GI_50_≥1 μM). ER: estrogen receptor; PR: progesterone receptor; Her2: Human Epidermal Growth Factor Receptor 2.
(DOCX)

S4 Table Efficacy of ARQ 092 in a panel of endometrial PDX models. ARQ 092 was tested for efficacy in a panel of 23 endometrial PDX models. For TGI analysis percent tumor growth inhibition (%TGI) values were calculated for each treatment group (T) versus control (C) and reported as percentage change in tumor volume. The PDX models are annotated for AKT1, AKT2, PIK3CA, PTEN, TSC1/2 and KRAS mutations. * Sequence information is not available.
(DOCX)
