# Supplementary figures and images for: Targeting AKT1-E17K and the PI3K/AKT Pathway with an Allosteric AKT Inhibitor, ARQ 092
Source: PLoS One. 2015 Oct 15;10(10):e0140479. doi: 10.1371/journal.pone.0140479 (PMC4607407; doi:10.1371/journal.pone.0140479)

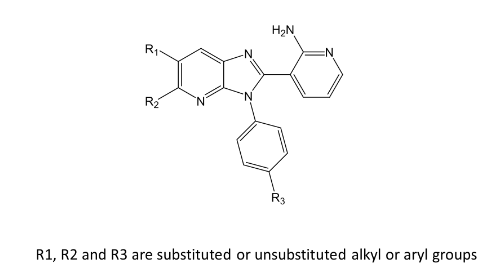

Supplement: S1 Fig — (TIF) [file pone.0140479.s001.tif]

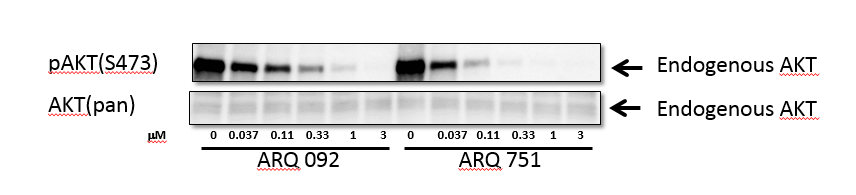

Supplement: S2 Fig — Endogenous WT AKT phosphorylation status was assessed in the same 293T cells transiently transfected with AKT-E17K-GFP. Both ARQ 092 and ARQ 751 inhibit pAKT. (TIF) [file pone.0140479.s002.tif]

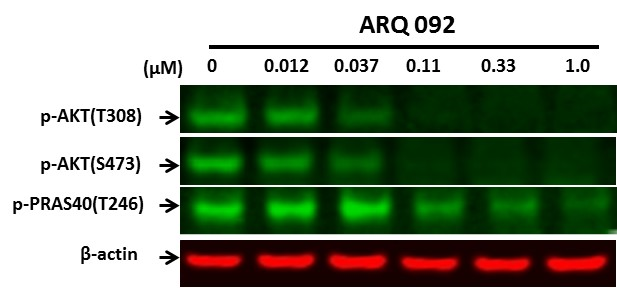

Supplement: S3 Fig — AN3CA cells were treated with various concentrations of ARQ 092 for two hours and stimulated with 100 ng/ml EGF and 100 nM insulin for 15 minutes. P-AKT(T308) and (S473) and phosphorylation of its downstream substrate PRAS40 were assessed by western blot analysis. The IC50 was determined for p-AKT(T308) and (S473) and p-PRAS40(T246). (TIF) [file pone.0140479.s003.tif]

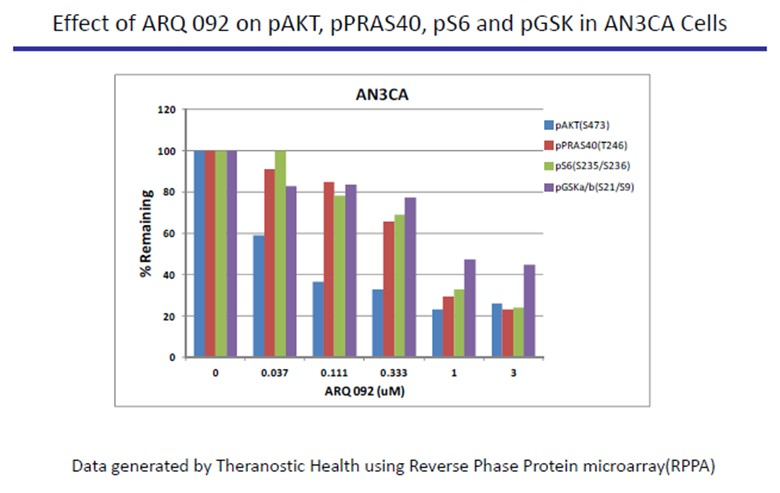

Supplement: S4 Fig — AN3CA cells were treated with ARQ 092 at 0, 0.03, 0.1, 0.3, 1, and 3 uM for 2 hours (n = 1). pAKT(S473), pPRAS40(T246), pS6(S235/S236) and pGSK3a/b(S21/S9) were assessed using RPMA (Theranostics Health). (TIF) [file pone.0140479.s004.tif]

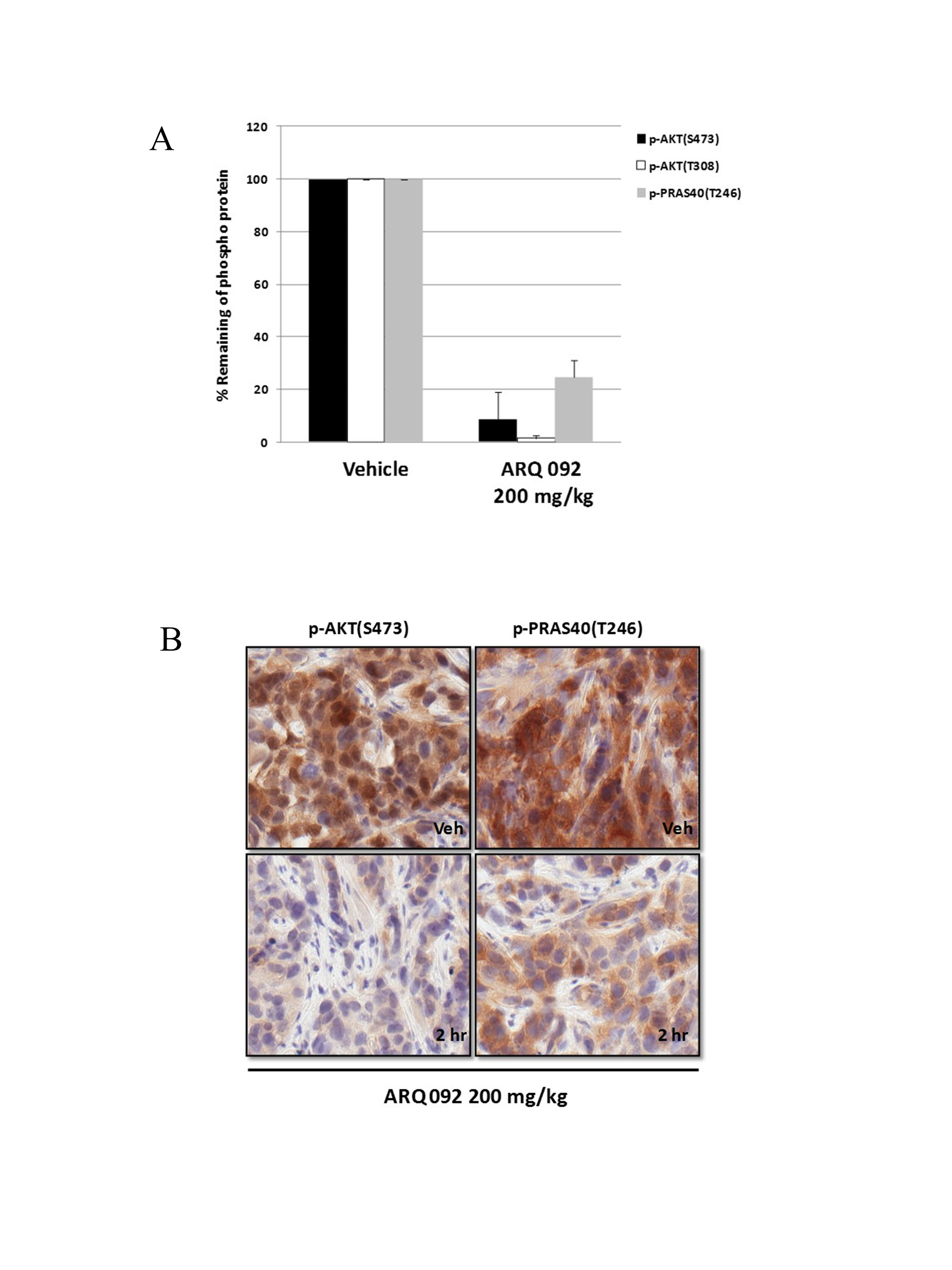

Supplement: S5 Fig — A: p-AKT (S473) and (T308), and p-PRAS40(T246) were assessed by western blot analysis from tumor tissues from and BT474 mouse xenograft after treatment with ARQ 092 at 200 mg/kg. The percentage remaining of the phospho proteins is shown and the vehicle group was designated as 100%. B: The same tumor samples were assessed by IHC for p-AKT(S473) and p-PRAS40(T246). Veh: Vehicle. (TIF) [file pone.0140479.s005.tif]

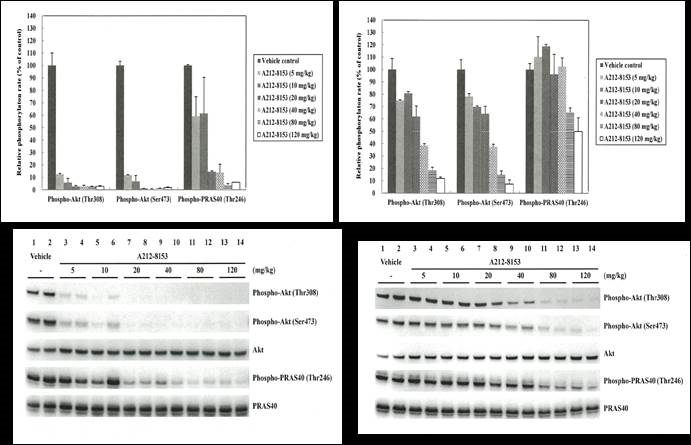

Supplement: S6 Fig — Inhibitory effect of ARQ 751 on AKT signaling pathway in AN3CA mouse xenografts at single dose levels of 5, 10, 20, 40, 80, and 120 mg/kg. Left panels show p-AKT and p-PRAS levels at 6 hours while the right panels show these levels at 24 hours. Note: A212-8153 = ARQ 751. (TIF) [file pone.0140479.s006.tif]

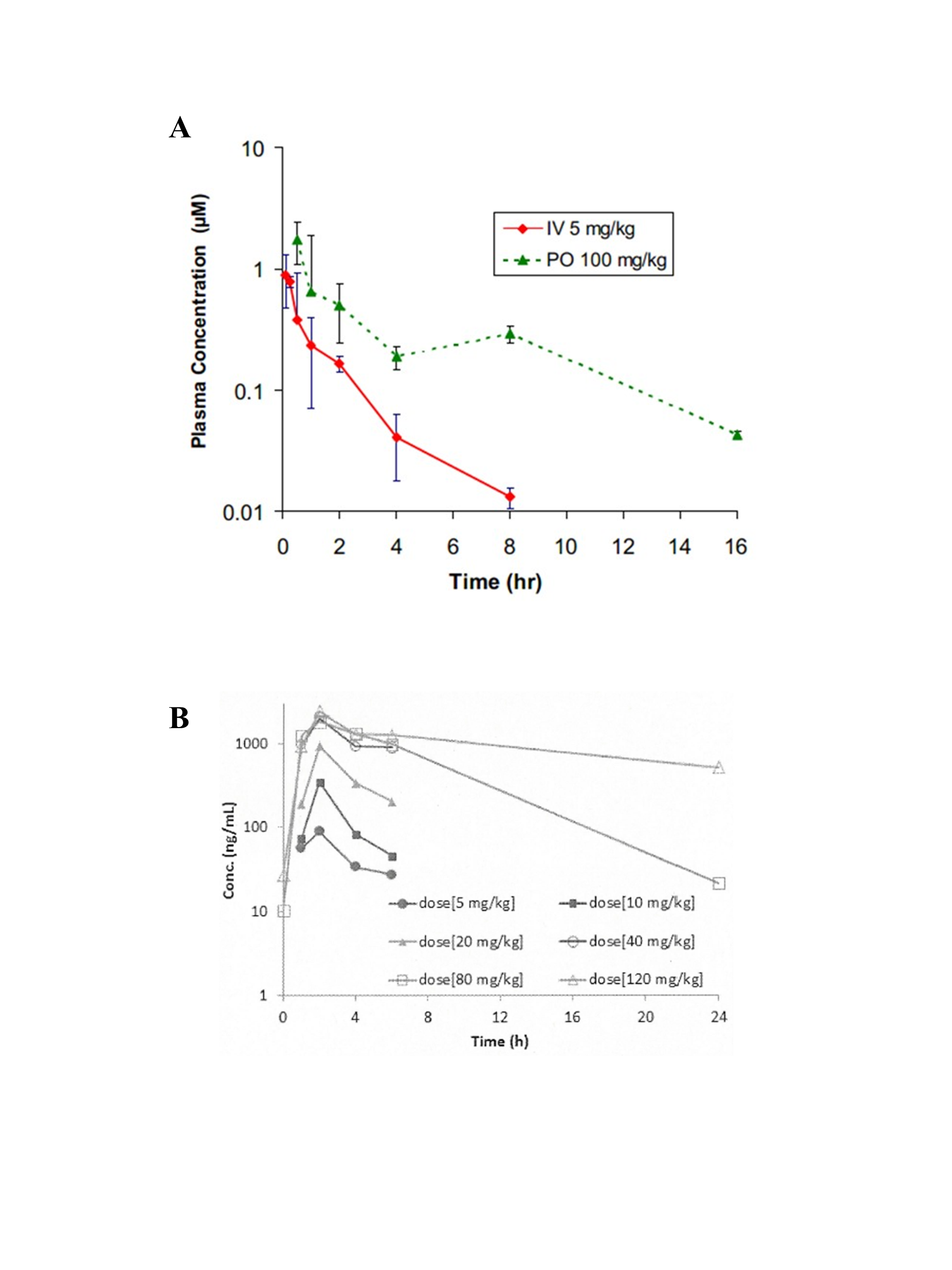

Supplement: S7 Fig — A: AN3CA xenograft mice were dosed iv at 5 mg/kg or PO with 100 mg/kg with ARQ 092. Blood samples were collected at 5, 15, and 30 minutes, and 1, 2, 4, and 8 hours post IV dose; 30 minutes, 1, 2, 4, 8, and 16 hours post PO dose. Blood samples were centrifuged and the plasma was collected. Plasma samples were analyzed by LC/MS/MS. Plasma concentration verse time profiles and PK parameters were determined following 5 mg/kg single IV and 100 mg/mg single PO ARQ 092 administration to mice. B: AN3CA xenograft mice were treated orally with ARQ 751 at dose levels of 5, 10, 20, 40, 80 and 120 mg/kg. Blood samples were collected at 0, 1, 2, 4, 6, 24 hours post-dose of ARQ 751 for measurement of the plasma concentrations. ARQ 751 plasma concentrations were determined using LC/MS/MS. (TIF) [file pone.0140479.s007.tif]

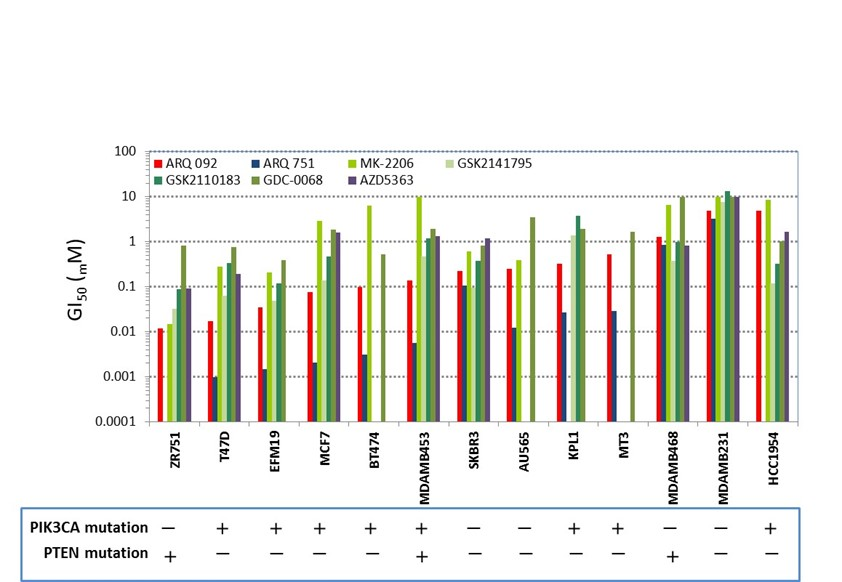

Supplement: S8 Fig — Comparison of the GI50 of the leading AKT inhibitors against a panel of breast cancer cell lines demonstrates that ARQ 092 is more potent or equally potent compared to leading AKT inhibitors while ARQ 751 is the most potent in 9 out of 13 breast cancer cell lines. (TIF) [file pone.0140479.s008.tif]
