# Supplementary material for: Targeting AKT1-E17K and the PI3K/AKT Pathway with an Allosteric AKT Inhibitor, ARQ 092
Source: PLoS One. 2015 Oct 15;10(10):e0140479. doi: 10.1371/journal.pone.0140479 (PMC4607407; doi:10.1371/journal.pone.0140479)
Supplement: S1 Methods — (DOCX) [file pone.0140479.s009.docx]

**Supplemental Methods**

**Reagents**

No additional reagents other than those identified in the main manuscript were used to generate supplemental data.

**Enzyme-linked Immunosorbent Assay (ELISA)**

AN3CA cells were treated with ARQ 751 at concentrations of 2000, 500, 125, 31.3, 7.81, 1.95, 0.488, and 0.122 nM (n = 6) for 1 hour and then fixed with methanol, quenched, and blocked. P-AKT (Thr308), p-AKT (Ser473) and p-PRAS40 (Thr246) in the cells were probed with corresponding rabbit antibodies (Cell Signaling Technology). Total AKT and total PRAS40 in the cells were probed with corresponding mouse antibodies (Cell Signaling Technology). Horseradish peroxidase (HRP)-conjugated goat anti-rabbit IgG (Cell Signaling Technology) and alkaline phosphatase (AP)-conjugated goat anti-mouse IgG (Santa Cruz Biotechnology, Inc., Dallas, TX) were used as secondary antibodies. The HRP chemiluminescent detection was performed with SuperSignal ELISA Pico Chemiluminescent Substate (Thermo Fisher Scientific, Inc.) and PHERAstar (BMG Labtech Inc., Cary, NC) according the the manufacturer’s instructions. After washing the AP chemiluminescent detection was performed with ELISA-Light Immunoassay System with CDP-Star and Sapphire-II Substrate/Enhancer Solution (Life Technologies Corporation) and PHERAstar according to the manufacturer’s instructions. The chemiluminescent signal was rounded to an integer in MARS 2.40 (BMG Labtech, Inc.) and the relative phosphorylation rate was calculated.

**Reverse Phase Protein Analysis (RPPA)**

AN3CA cells were plated in 6-well plate and then treated with various concentration of various concentrations of ARQ 092 for 2 hrs. The cell pellet was collected and snap frozen and shipped to Theranostics Health ([www.theranosticshealth.com](http://www.theranosticshealth.com)). p-AKT(S473), p-PRAS40(T246), p-S6 (S235/S236) and p-GSK3ab(S21/S9) levels were assessed using Reverse Phase Protein Array.

**Cell proliferation MTS assays and GI_50_ determination**

For cell proliferation MTS assay, cells were seeded at an optimal number per well in 130 µl of full growth media in 96-well tissue culture plates, incubated overnight and then treated with compounds at a starting concentration of 33.3 or 100 µM. Treated cells were incubated at 37°C for 72 hours in 5% CO_2_.

Thirty microliters of MTS reagent (18.4 mg/ml) combined with PMS (0.92 mg/ml) at a ratio of 20:1 were added to each well, and the plates were incubated at 37°C for 4 hours in 5% CO_2_. The absorbance was measured at 490 nM using a Victor microplate reader (Perkin Elmer, Waltham, MA).

The GI_50_ was then determined using Microsoft ExcelFit software in Activity Base.

**PDX endometrial tumor models**

A panel of 23 Patient-derived xenograft (START-PDX) endometrial tumor models were screened for ARQ 092 efficacy. The tumors were established from viable human tumor tissue or fluid and have been serially passaged in immunocompromised mice. Pre-study tumor volumes were recorded for each experiment beginning approximately one week prior to its estimated start date. When tumors reached the appropriate Tumor Volume Initiation (TVI) range (150-300 mm^3^), animals were randomized into treatment and control groups (n = 2) and ARQ 092 was dosed once daily orally at 100 mg/kg for 14 days.

**ARQ 092 PK Analysis**

Twenty-one and 18 female nude mice (NCr) were used in this study for IV and oral

dosing of ARQ 092, respectively. The mice were sacrificed and blood samples were collected at 5, 15, and 30 minutes, and 1, 2, 4, and 8 hours post IV dose; 30 minutes, 1, 2, 4, 8, and 16 hours post PO dose. Blood samples were centrifuged and the plasma was collected. Plasma samples were analyzed by LC/MS/MS. Plasma concentration verse time profiles and PK parameters were determined following 5 mg/kg single IV and 100 mg/mg single PO ARQ 092 administration to mice.

**ARQ 751 PK Analysis**

AN3CA xenograft mice were treated orally with ARQ 751 at dose levels of 5, 10, 20, 40, 80 and 120 mg/kg. Blood samples were collected at 0, 1, 2, 4, 6, 24 hours post-dose of ARQ 751 for measurement of the plasma concentrations. ARQ 751 plasma concentrations were determined using LC/MS/MS. Plasma concentration versus time profiles were generated using the plasma concentrations determined from the LC/MS/MS analysis.

**Methods not specified here which were used to generate the data presented in the supplementary section were already described in the main manuscript.**
