# Supplementary material for: Targeting AKT1-E17K and the PI3K/AKT Pathway with an Allosteric AKT Inhibitor, ARQ 092
Source: PLoS One. 2015 Oct 15;10(10):e0140479. doi: 10.1371/journal.pone.0140479 (PMC4607407; doi:10.1371/journal.pone.0140479)
Supplement: S1 Table — The inhibitory activity of ARQ 751 on the Akt signaling pathway in the human endometrial cancer AN3 CA cell line using cell-based ELISA. The EC50 values for the inhibition of p-Akt (Thr308), p-Akt (Ser473), and p-PRAS40 (Thr246) were 5, 10, and 49 nM, respectively. (DOCX) [file pone.0140479.s010.docx]

|  |  | **95% CI** | |
| --- | --- | --- | --- |
| **Target** | **EC_50_ (nM)** | **Lower** | **Upper** |
| p-AKT(T308) | 5.10 | 4.76 | 5.49 |
| p-AKT(S473) | 10.20 | 8.30 | 12.86 |
| p-PRAS40(T246) | 48.55 | 42.00 | 56.91 |
