# Supplementary material for: Targeting AKT1-E17K and the PI3K/AKT Pathway with an Allosteric AKT Inhibitor, ARQ 092
Source: PLoS One. 2015 Oct 15;10(10):e0140479. doi: 10.1371/journal.pone.0140479 (PMC4607407; doi:10.1371/journal.pone.0140479)
Supplement: S2 Table — Select cancer cell lines were treated with various concentrations of ARQ 092, ARQ 751, MK-2206, or GDC-0068 for single agent. IC50 was calculated using Activity Base. Data shown as mean ± standard deviation. MDA-MB-453: 3000 cells; NCI-H1650: 2000; and KU-19-19: 2500 cells. (DOCX) [file pone.0140479.s011.docx]

|  | **IC_50_ (µM)** | | |
| --- | --- | --- | --- |
| **AKT Inhibitor** | **MDA-MB-453** | **NCI-H1650** | **KU-19-19** |
|  | (PIK3CAH1047R) | (PTEN null) | (AKT1-E17K, NrasQ61R) |
| ARQ 092 | 0.12±0.06 | 6.83±1.38 | 12.4±2.4 |
| GDC-0068 | 1.00±0.16 | >33.33 | 55.0±5.1 |
| MK-2206 | 0.26±0.02 | 15.2±3.01 | 21.5±5.3 |
| ARQ 751 | 0.01±0.005 | 11.0±0.63 | 17.3±1.6 |
