# Supplementary material for: Targeting AKT1-E17K and the PI3K/AKT Pathway with an Allosteric AKT Inhibitor, ARQ 092
Source: PLoS One. 2015 Oct 15;10(10):e0140479. doi: 10.1371/journal.pone.0140479 (PMC4607407; doi:10.1371/journal.pone.0140479)
Supplement: S3 Table — The correlation of PIK3CA/PIK3R1 mutation with A: ARQ 092 and B: ARQ 751 sensitivity in breast cancer cell lines was determined as shown on this table. Seven out of 8 (88%) cell lines bearing PIK3CA/PIK3R1 mutations are sensitive to ARQ 092 (GI50<1 μM). Among all breast cancer cell lines tested, 11 out of 18 (61%) cell lines are sensitive to ARQ 092 while triple negative breast cancer cell lines are resistant (GI50≥1 μM). ER: estrogen receptor; PR: progesterone receptor; Her2: Human Epidermal Growth Factor Receptor 2. (DOCX) [file pone.0140479.s012.docx]

| **Breast Cancer Cell Lines** | **GI_50_(µM)** | **PIK3CA** | **ER** | **PR** | **HER2** |
| --- | --- | --- | --- | --- | --- |
| T47D | 0.02 | H1047R | + | + | - |
| EFM-19 | 0.04 | H1047R | + | + | - |
| MCF-7 | 0.08 | E545K | + | + | - |
| BT474 | 0.10 | K111N | + | + | + |
| MDA-MB-453 | 0.14 | H1047R | - | - | + |
| SK-BR-3 | 0.22 | - | - | - | + |
| CAMA-1 | 0.23 | - | + | + | - |
| AU565 | 0.25 | - | - | - | + |
| KPL-1 | 0.33 | E545K | + | + | - |
| MT-3 | 0.52 | unknown | unknown | unknown | unknown |
| BT-20 | 0.78 | H1047R, P539R | - | - | - |
| Hs 578T | 1.03 | - | - | - | - |
| MDA-MB-468 | 1.30 | - | - | - | - |
| MX1 | 1.94 | unknown | unknown | unknown | unknown |
| BT-549 | 2.09 | - | - | - | - |
| MDA-MB-231 | 4.82 | - | - | - | - |
| MDA-MB-436 | 5.86 | - | - | - | - |
| BT-483 | 40.0 | E542K | + | + | - |

**A**

**B**

| **Breast Cancer Cell Lines** | **GI_50_(nM)** | **PIK3CA** | **ER** | **PR** | **HER2** |
| --- | --- | --- | --- | --- | --- |
| T47D | 1.05 | H1047R | + | + | - |
| EFM-19 | 1.54 | H1047R | + | + | - |
| MCF-7 | 2.20 | E545K | + | + | - |
| BT474 | 3.25 | K111N | + | + | + |
| MDA-MB-453 | 6.05 | H1047R | - | - | + |
| CAMA-1 | 9.30 | - | + | + | - |
| AU565 | 12.89 | - | - | - | + |
| KPL-1 | 28.74 | E545K | + | + | - |
| MT-3 | 31.17 | unknown | unknown | unknown | unknown |
| SK-BR-3 | 113.05 | - | - | - | + |
| Hs 578T | 148.98 | - | - | - | - |
| BT-549 | 723.75 | - | - | - | - |
| BT-20 | 730.09 | H1047R, P539R | - | - | - |
| MDA-MB-468 | 886.46 | - | - | - | - |
| MX1 | 1975.78 | unknown | unknown | unknown | unknown |
| MDA-MB-231 | 3412.71 | - | - | - | - |
| MDA-MB-436 | 6931.09 | - | - | - | - |
