# Supplementary material for: Targeting AKT1-E17K and the PI3K/AKT Pathway with an Allosteric AKT Inhibitor, ARQ 092
Source: PLoS One. 2015 Oct 15;10(10):e0140479. doi: 10.1371/journal.pone.0140479 (PMC4607407; doi:10.1371/journal.pone.0140479)
Supplement: S4 Table — ARQ 092 was tested for efficacy in a panel of 23 endometrial PDX models. For TGI analysis percent tumor growth inhibition (%TGI) values were calculated for each treatment group (T) versus control (C) and reported as percentage change in tumor volume. The PDX models are annotated for AKT1, AKT2, PIK3CA, PTEN, TSC1/2 and KRAS mutations. * Sequence information is not available. (DOCX) [file pone.0140479.s013.docx]

| **PDX model** | **Tumor Volume Mean** | **Std. Err. Mean** | **% T/C** | **PIK3CA** | **AKT1** | **AKT2** | **PIK3R1** | **PTEN** | **TSC1** | **TSC2** | **KRAS** |
| --- | --- | --- | --- | --- | --- | --- | --- | --- | --- | --- | --- |
| ST189 | 189 | 111 | -15% | WT | WT | WT | WT | E235X- stop codon | WT | G440S | WT |
| ST1619 | 261 | 6 | 0% | WT | WT | WT | WT | WT | M271T | L604P | WT |
| ST1529 | 235 | 31 | 0% | I391M | WT | WT | M56I | WT | WT | WT | WT |
| ST259 | 325 | 247 | 9% | R93W, D350G | WT | WT | WT | R130G | WT | WT | WT |
| ST993* | 300 | 0 | 10% | UNK | UNK | UNK | UNK | UNK | UNK | UNK | UNK |
| ST040 | 229 | 51 | 13% | WT | WT | WT | WT | WT | WT | WT | WT |
| ST1385 | 256 | 77 | 23% | WT | WT | D161A | WT | R130X- stop codon | WT | L604P | WT |
| ST328 | 313 | 109 | 26% | R108H, Q546L | WT | WT | WT | WT | WT | P1174L | WT |
| ST1061 | 440 | 236 | 28% | WT | E17K | WT | WT | WT | WT | WT | WT |
| ST413 | 234 | 66 | 35% | WT | WT | WT | WT | WT | WT | I912I, S1316R, Q1487E | WT |
| ST357 | 632 | 60 | 39% | WT | WT | WT | M26I | WT | WT | WT | G12A |
| ST657 | 523 | 101 | 46% | E545A | WT | WT | WT | WT | WT | WT | WT |
| ST819 | 229 | -- | 49% | E726K | WT | WT | K447Q | R130Q | WT | WT | WT |
| ST1006 | 623 | 51 | 55% | WT | WT | WT | WT | R130Q | M322T | R367Q | WT |
| ST633 | 2900 | 1230 | 70% | Q546K | WT | WT | WT | WT | WT | WT | WT |
| ST609 | 953 | 277 | 73% | WT | WT | WT | WT | R130X- stop codon | WT | WT | WT |
| ST1152 | 2422 | 433 | 75% | WT | WT | WT | WT | WT | WT | WT | WT |
| ST013 | 540 | 8 | 100% | WT | WT | WT | M56I | WT | WT | WT | WT |
| ST477 | 225 | 75 | 100% | WT | WT | WT | M56I | WT | M322T | WT | WT |
| ST1094 | 394 | 28 | 100% | WT | WT | WT | WT | WT | WT | WT | WT |
| ST1764 | 896 | 78 | 100% | WT | WT | WT | WT | G132V | L690P, M322T | WT | WT |
| ST404 | 632 | 60 | Not Calculated | WT | WT | WT | WT | WT | H731Y, K586R | A289V | WT |
| ST953 | 150 | -- | Not Calculated | I391M | WT | WT | M326I | WT | WT | WT | WT |
